# Rapid evolution of Mexican H7N3 highly pathogenic avian influenza viruses in poultry

**DOI:** 10.1371/journal.pone.0222457

**Published:** 2019-09-12

**Authors:** Sungsu Youk, Dong-Hun Lee, Helena L. Ferreira, Claudio L. Afonso, Angel E. Absalon, David E. Swayne, David L. Suarez, Mary J. Pantin-Jackwood

**Affiliations:** 1 Exotic and Emerging Avian Viral Diseases Research Unit, Southeast Poultry Research Laboratory, U.S. National Poultry Research Center, Agricultural Research Service, U.S. Department of Agriculture, Athens, Georgia, United States of America; 2 Department of Pathobiology & Veterinary Science, University of Connecticut, Storrs, Mansfield, Connecticut, United States of America; 3 University of Sao Paulo, ZMV- FZEA, Pirassununga, Brazil; 4 Instituto Politécnico Nacional, Centro de Investigación en Biotecnología Aplicada, Tlaxcala, México; Istituto Zooprofilattico Sperimentale delle Venezie, ITALY

## Abstract

Highly pathogenic avian influenza (HPAI) virus subtype H7N3 has been circulating in poultry in Mexico since 2012 and vaccination has been used to control the disease. In this study, eight Mexican H7N3 HPAI viruses from 2015–2017 were isolated and fully sequenced. No evidence of reassortment was detected with other avian influenza (AI) viruses, but phylogenetic analyses show divergence of all eight gene segments into three genetic clusters by 2015, with 94.94 to 98.78 percent nucleotide homology of the HA genes when compared to the index virus from 2012. The HA protein of viruses from each cluster showed a different number of basic amino acids (n = 5–7) in the cleavage site, and six different patterns at the predicted N-glycosylation sites. Comparison of the sequences of the Mexican lineage H7N3 HPAI viruses and American ancestral wild bird AI viruses to characterize the virus evolutionary dynamics showed that the nucleotide substitution rates in PB2, PB1, PA, HA, NP, and NS genes greatly increased once the virus was introduced into poultry. The global nonsynonymous and synonymous ratios imply strong purifying selection driving the evolution of the virus. Forty-nine positively selected sites out of 171 nonsynonymous mutations were identified in the Mexican H7N3 HPAI viruses, including 7 amino acid changes observed in higher proportion in North American poultry origin AI viruses isolates than in wild bird-origin viruses. Continuous monitoring and molecular characterization of the H7N3 HPAI virus is important for better understanding of the virus evolutionary dynamics and further improving control measures including vaccination.

## Introduction

Avian influenza (AI) viruses belong to the family Orthomyxoviridae, genus *Influenzavirus A*, and contain a negative-sense, eight segmented RNA genome (Polymerase basic 2, PB2; Polymerase basic 1, PB1; Polymerase acid, PA; Hemagglutinin, HA; Nucleocapsid protein, NP, Neuraminidase, NA; Matrix, M; Nonstructural protein, NS) [1]. Genetic features and/or severity of disease in chickens determine whether the virus is classified as low pathogenicity avian influenza (LPAI) or high pathogenicity avian influenza (HPAI) virus [2]. Multiple substitutions or insertions of basic amino acids in the cleavage site of the HA of H5 and H7 subtypes of LPAI viruses allow them to be cleaved by common cellular proteases, and the mutated virus can spread systemically in a host causing the highly pathogenic phenotype [3]. AI viruses have a high error rate during the transcription of the viral genome because of a relatively low RNA polymerase fidelity, providing an opportunity for the virus to evolve and adapt to new environments [4, 5]. These changes might be associated with adaptation for optimal replication and transmission in a new host or escaping an immune pressure caused by a previous infection with other AI viruses or by vaccination.

There have been four unrelated outbreaks of H7N3 subtype HPAI in poultry in the American continents, including outbreaks in Chile, Canada, and Mexico [6–9]. The Mexican H7N3 HPAI virus has been causing outbreaks in commercial chickens and backyard poultry in Mexico since its first detection in 2012 [10]. Although a direct LPAI precursor for the Mexican H7N3 HPAI virus was not identified during the 2012 initial outbreak, phylogenetic analysis support that the outbreak was caused by a LPAI virus introduced from wild aquatic birds into poultry and subsequently acquiring additional basic amino acids in the HA cleavage site generating the highly pathogenic phenotype [9, 11, 12]. The LPAI virus originated from the large North America LPAI virus pool through reassortment events, with different virus segments contributed by wild waterfowl from different North American flyways; HA, NA, NP, M, and NS from wild birds migrating along the central flyway, and PB2, PB1 and PA introduced via the western flyway (12). To control the disease in poultry and reduce the risk to human health, a vaccination campaign, along with stamping out and biosecurity, was implemented in Mexico and are still in place. Despite the control measures undertaken, cases of H7N3 HPAI in poultry have been repeatedly documented and two confirmed cases of human infection were reported from poultry workers [13, 14].

In previous studies, we demonstrated phenotypic and antigenic changes in recent Mexican lineage H7N3 viruses. A H7N3 HPAI virus isolated in 2016 was less adapted to mallards than the index virus from 2012, and increase in N-glycosylation on the HA was associated with escape of a field virus from a older vaccine [15, 16]. However, detailed information on the evolutionary history of the Mexican lineage H7N3 HPAI virus is lacking. In the present study, we investigated the genetic diversity and the evolutionary dynamics of the H7N3 HPAI viruses using complete genome sequencing and comparative phylogenetic approaches to compare the H7N3 HPAI viruses with its ancestral wild bird-origin AI viruses.

## Materials and methods

### Virus isolation and genome sequencing

Eight Mexican lineage H7N3 HPAI viruses were sequenced in this study. The viruses were isolated from oral swabs obtained from backyard chickens located close to commercial farms. The samples were processed in the Instituto Politécnico Nacional, Centro de Investigación en Biotecnología Aplicada, Tlaxcala, Mexico. All the procedures for virus detection and virus isolation in chicken embryonating eggs were approved by the Care and Use of Animals Committee of the Instituto Politécnico Nacional. Birds were not euthanized for sampling. Twelve out of 48 samples resulted positive by standard embryonating egg inoculation, and eight of these samples were selected for sequencing. Four viruses were collected in 2015: two from backyard chickens in the state of Puebla, one from a fighting cock in the state of Oaxaca, and one from a backyard chicken in the state of Jalisco. Four viruses were collected in 2017 from backyard chickens in the state of Jalisco. Viral RNA was extracted from the samples by using the QIAamp viral RNA kit (QIAGEN) and shipped to the Southeast Poultry Research Laboratory (SEPRL) USDA, GA, USA. RNA samples were reverse-transcribed and DNA libraries for next-generation sequencing were prepared as described previously [17]. The Nextera XT DNA Sample Preparation Kit (Illumina, CA, USA) was used to generate multiplexed paired-end sequencing libraries, according to the manufacturer's instructions. The sequences were analyzed and assembled using the Geneious 10.0.9 software [18] or MIRA version 3.4.1 within a customized workflow on the Galaxy platform as previously described [19]. The sequences have been uploaded in GenBank with the collection dates (S1 Table).

### Viral sequences used in this study

All available North American AI virus sequences related to the initial Mexican H7N3 viruses (A/chicken/Jalisco/CPA1/2012) identified during 2000–2018 were searched by using the BLAST function and classified by segment (access date: July 2018, number of output: 500) [20]. The identical sequences were removed by using Geneious version 10.2.1, and other HPAI sequences were excluded from the dataset. The numbers of sequences used for phylogenetic analysis are as follow: PB2 (n = 393), PB1 (n = 350), PA (n = 376), HA (n = 372), NP (= 358), NA (n = 398), M (n = 343), and NS (n = 305). In addition to the viruses sequenced in this study, full genome sequences of 18 viruses available in the Influenza Research Database, including viruses previously sequenced in our laboratory were retrieved (access date: July 2018) for full genome analysis of the Mexican H7N3 viruses from 2012 to 2017 [15]. For the estimations of substitution rate using the Bayesian analyses, the wild bird AI sequences clustering with the initial Mexican H7N3 sequences were extracted from the Maximum-likelihood phylogenetic trees, with bootstrap value (n > 70).

For the selection analyses of the Mexican H7N3 HPAI viruses among poultry and wild birds, AI genome sequences were downloaded from the Influenza Research Database (Search parameters: Date range: none, Host: Avian, Geographic grouping: North America), reduced to non-redundant dataset with 100% cut-off value, and classified into two categories by its host species; the poultry category includes sequences from chicken, ostrich, quail, and turkey, and wild birds category includes sequences from all avian isolates except the poultry species. The total number of sequences used for the comparison were as follows: PB2 (n = 8160), PB1 (n = 8434), PA (n = 8228), H7 HA (n = 881), N3 NA (n = 859), M (n = 6018), and NS (n = 4291).

### Maximum-likelihood phylogenetic analysis

Maximum-likelihood (ML) phylogenies of each segment were generated by using RAxML and the general time-reversible nucleotide substitution model, with 1,000 rapid bootstrap replicates [21]. To calculate the evolutionary rate of global AI viruses in North America, an estimate of nucleotide substitution rate was obtained using a root-to-tip regression method available in the TempEst v1.5.1 program [22] in which genetic distances estimated from a maximum likelihood trees are plotted against date of sampling. The global substitution rates of North American AI viruses were obtained from the slopes of the regression line.

### Bayesian phylogenetic analysis

Selection of the best-fit nucleotide substitution model for each segment with Akaike Information Criterion (AIC) was performed with jModelTest 2.1.9 [23], and the nucleotide substitution models for each segment were used for Bayesian phylogenetic analysis: GTR + G for PB2, NP, M, and HA; GTR + I for NA; GTR + I + G for PB1 and PA; HKY + I for NS. Bayesian relaxed clock phylogenetic analysis of each gene was performed, and the time of the Most Recent Common Ancestor (TMRCA) of the H7N3 viruses was estimated using BEAST v1.8.4 program [24]. For the TMRCA estimation, we applied an uncorrelated log-normal distribution relaxed clock method, the selected nucleotide substitution model of each segment, the coalescent GMRF Bayesian Skyride model [25]. Three independent Markov chain Monte Carlo (MCMC) analyses were run for 50 million generations with sampling every 5,000 steps and a burn-in of 5 million states, and then combined and checked for convergence by high ESS values (>200) in Tracer.

To compare the nucleotide substitution rates before and after the introduction of the Mexican H7N3 HPAI into poultry, we used the H7N3 HPAI viruses and their ancestral AI sequences identified in wild birds. We performed Bayesian random local clock (RLC) analysis of each gene as the RLC model allows estimation of the nucleotide substitution rate-change across the tree [26]. To avoid the influence of transient deleterious mutations on external branches of phylogeny, we estimated mean substitution rates for internal branches. All internal branch lengths and rates were extracted using TreeStat program v1.8.4, after exclusion of initial 1000 burn-in trees. Assuming that heights of the first internal nodes for all H7N3 HPAI viruses are the time of the first introduction to the Mexican poultry, all the nucleotide substitution rates were divided into two separate terms; the rates before the first internal nodes represent viral evolution in wild bird, and the rate after the first internal nodes represent viral evolution in the Mexican poultry.

To visualize the shift of rate variation of the hemagglutinin (HA) and neuraminidase (NA) gene as a process in time, we partitioned time before the last sequence sampling date into 82 bins for HA and 97 bins for NA. For each bin, we constructed the empirical posterior density of relative rates active along the tree during that time period. Discretizing the branch length, paring them with the rate were performed using R statistical software package [27]. The heat map with the posterior density of relative rates were generated with Graph Pad Prism software 7.05 (GraphPad Software, San Diego, CA).

### Analysis of selection pressures and potential HA and NA N-glycosylation

Positively selected sites were estimated by implementing four different codon-based maximum likelihood approaches on datamonkey server (http://www.datamonkey.org/) using the same dataset used for the Bayesian analysis, which includes the Mexican H7N3 viruses and wild bird AI sequences clustering with the initial Mexican H7N3 sequences: single likelihood ancestor counting (SLAC), fixed effects likelihood (FEL), mixed effects model of episodic (MEME), and fast unconstrained Bayesian approximation (FUBAR). In addition, amino acid sites under positive selection in each segment were determined using the Renaissance counting method implemented in BEAST package v1.8.4 [28]. The number of dN/dS for each state on each branch of Bayesian phylogeny was recorded and then converted into dN/dS values [29]. The potential N-glycosylation sites for the HA and Neuraminidase (NA) protein of the Mexican H7N3 viruses were predicted using NetNGlyc server 1.0 [30].

### Statistical analysis

A comparison of the H7N3 evolutionary rates between before and after the introduction into poultry in Mexico was performed using the probability density function of posterior simulated data for each population mean rate obtained using BEAST. Assuming a normal distribution of mean substitution rates with a standard deviation derived from the 95% highest probability density (HPD) values, we calculated the probability whether the distributions of the evolutionary rates from viruses before the introduction of the H7N3 in poultry were lower than those of rates from viruses sampled after introduction or not. A probability of > 0.90 was evaluated relevant.

## Results

### Phylogenetic analysis and HA cleavage sites

**Fig 1:**
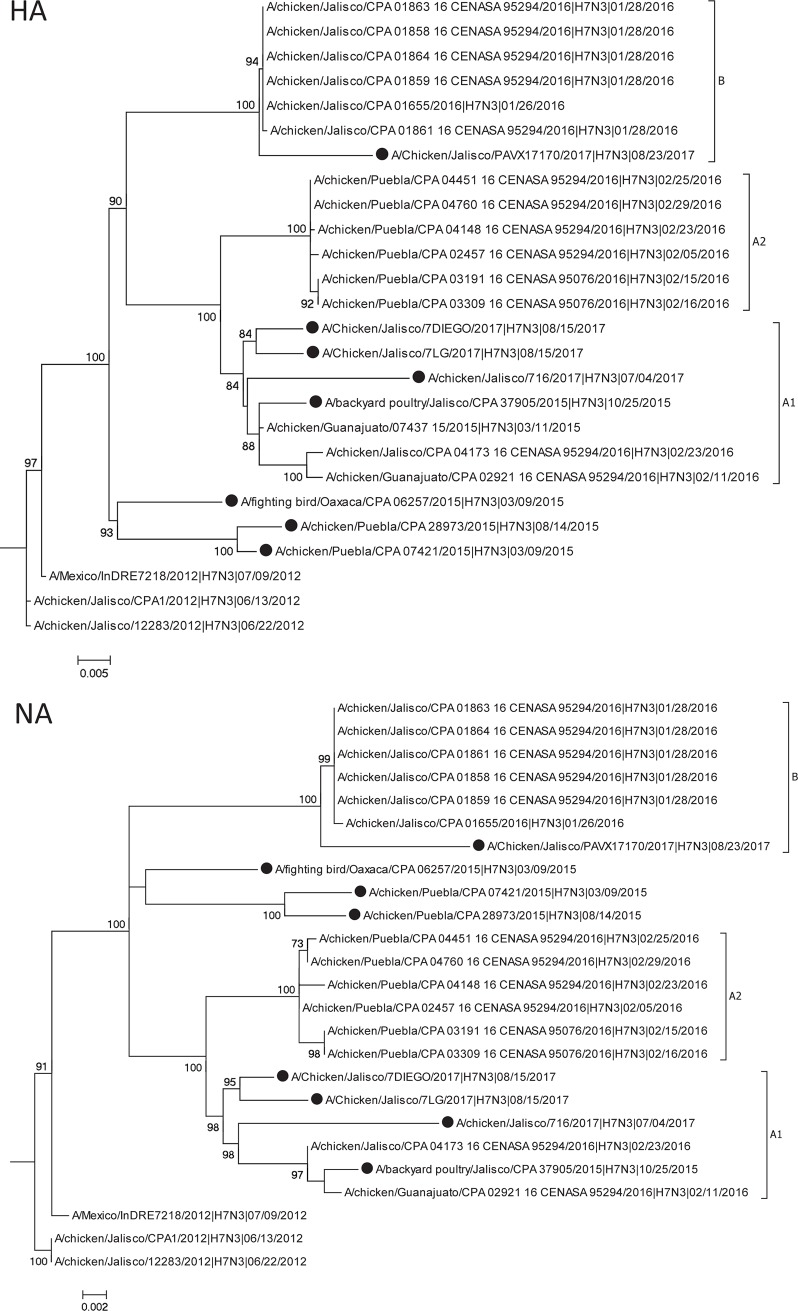
Maximum-likelihood phylogeny of HA and NA gene of the Mexican H7N3 HPAI viruses. The HA gene of the Mexican H7N3 HPAI viruses were divided into three genetic clusters (A1, A2, and B). The closed circles indicate the H7N3 viruses isolated and sequenced in this study.

The maximum-likelihood phylogenetic analysis showed that the HA and NA gene of the Mexican H7N3 viruses diverged into two clusters (A and B), with three transitional isolates in 2015 (Fig 1). The cluster A further divided into two subclusters (A1 and A2). (S1 Fig). The three clusters tend to be constrained by regional geographic distribution; the viruses in cluster A1 were isolated in Jalisco and neighboring Guanajuato regions; the viruses in cluster A2 were from the Puebla region; and the viruses in cluster B were from the Jalisco region. The other genes (PB2, PB1, PA, NP, M, and NS) also showed the same clustering pattern as the HA gene. In terms of virus reasortment, there were no introductions of other AI virus genes into to the H7N3 viruses, but three isolates had reassorments with PB2, PB1, and NS genes in between three clusters (Table 1).

**Table 1:** Identification of cluster constellation and HA cleavage sites.

Strain	Segments								HA cleavage site ^a^
	PB2	PB1	PA	HA	NP	NA	M	NS	
Wild bird consensus	-	-	-	-	-	-	-	-	PENPK___________TR|GLF
A/chicken/Jalisco/CPA1/2012|H7N3|06/13/2012 –The first identification	-	-	-	-	-	-	-	-	PENPKD**RK**S**R**H**RR**T**R**|GLF
A/chicken/Jalisco/12283/2012|H7N3|06/22/2012	-	-	-	-	-	-	-	-	PENPKD**RK**S**R**H**RR**T**R**|GLF
A/fighting_bird/Oaxaca/CPA_06257/2015|H7N3|03/09/2015	-	-	-	-	-	-	-	-	PENPKD**RK**S**R**H**RR**T**R**|GLF
A/chicken/Puebla/CPA_07421/2015|H7N3|03/09/2015	-	-	-	-	-	-	-	-	PENSKDM**K**S**R**H**RK**T**R**|GLF
A/chicken/Puebla/CPA_28973/2015|H7N3|08/14/2015	-	-	-	-	-	-	-	-	PENSKDM**K**S**R**H**RK**T**R**|GLF
A/chicken/Guanajuato/07437_15/2015|H7N3|03/11/15—Vector vaccine strain	-	-	-	A1	-	-	-	-	PENPKD**RK**S**R**H**RR**T**R**|GLF
A/backyard_poultry/Jalisco/CPA_37905/2015|H7N3|10/25/2015	A1	A1	A1	A1	A1	A1	A1	A1	PENPKD**RKRR**H**RR**T**R**|GLF
A/chicken/Guanajuato/CPA_02921_16_CENASA_95294/2016|H7N3|02/11/2016	A1	A1	A1	A1	A1	A1	A1	A1	PENPKD**RK**S**R**H**RR**T**R**|GLF
A/chicken/Jalisco/CPA_04173_16_CENASA_95294/2016|H7N3|02/23/2016	A1	A1	A1	A1	A1	A1	A1	**A2**	PENPKD**RK**S**R**H**RR**T**R**|GLF
A/chicken/Jalisco/716/2017|H7N3|07/04/2017	A1	A1	A1	A1	A1	A1	A1	A1	PENPKDW**K**S**R**H**RR**T**R**|GLF
A/Chicken/Jalisco/7LG/2017|H7N3|08/15/2017	A1	A1	A1	A1	A1	A1	A1	A1	PENPKD**RK**S**R**H**RR**T**R**|GLF
A/Chicken/Jalisco/7DIEGO/2017|H7N3|08/15/2017	A1	A1	A1	A1	A1	A1	A1	A1	PENPKD**RK**G**R**H**RR**T**R**|GLF
A/chicken/Puebla/CPA_02457_16_CENASA_95294/2016|H7N3|02/05/2016	A2	A2	A2	A2	A2	A2	A2	A2	PENPKD**RK**N**R**H**RR**T**R**|GLF
A/chicken/Puebla/CPA_03191_16_CENASA_95076/2016|H7N3|02/15/2016	A2	A2	A2	A2	A2	A2	A2	A2	PENPKD**RK**N**R**H**RR**T**R**|GLF
A/chicken/Puebla/CPA_03309_16_CENASA_95076/2016|H7N3|02/16/2016	A2	A2	A2	A2	A2	A2	A2	A2	PENPKD**RK**N**R**H**RR**T**R**|GLF
A/chicken/Puebla/CPA_04148_16_CENASA_95294/2016|H7N3|02/23/2016	A2	**A1**	A2	A2	A2	A2	A2	A2	PENPKD**RK**N**R**H**RR**T**R**|GLF
A/chicken/Puebla/CPA_04451_16_CENASA_95294/2016|H7N3|02/25/2016	A2	A2	A2	A2	A2	A2	A2	A2	PENPKD**RK**N**R**H**RR**T**R**|GLF
A/chicken/Puebla/CPA_04760_16_CENASA_95294/2016|H7N3|02/29/2016	A2	A2	A2	A2	A2	A2	A2	A2	PENPKD**RK**N**R**H**RR**T**R**|GLF
A/chicken/Jalisco/CPA_01655/2016|H7N3|01/26/2016	B	B	B	B	B	B	B	B	PENPKG**KK**S**R**H**RK**T**R**|GLF
A/chicken/Jalisco/CPA_01858_16_CENASA_95294/2016|H7N3|01/28/2016	**A2**	B	B	B	B	B	B	B	PENPKG**KK**S**R**H**RK**T**R**|GLF
A/chicken/Jalisco/CPA_01863_16_CENASA_95294/2016|H7N3|01/28/2016	B	B	B	B	B	B	B	B	PENPKG**KK**S**R**H**RK**T**R**|GLF
A/chicken/Jalisco/CPA_01864_16_CENASA_95294/2016|H7N3|01/28/2016	B	B	B	B	B	B	B	B	PENPKG**KK**S**R**H**RK**T**R**|GLF
A/chicken/Jalisco/CPA_01859_16_CENASA_95294/2016|H7N3|01/28/2016	B	B	B	B	B	B	B	B	PENPKG**KK**S**R**H**RK**T**R**|GLF
A/chicken/Jalisco/CPA_01861_16_CENASA_95294/2016|H7N3|01/28/2016	B	B	B	B	B	B	B	B	PENPKG**KK**S**R**H**RK**T**R**|GLF
A/Chicken/Jalisco/PAVX17170/2017|H7N3|08/23/2017	B	B	B	B	B	B	B	B	PENPKG**RK**S**R**H**RK**T**R**|GLF

^a^ Underlined amino acids indicate multiple basic amino acid insertion site. Arginine (R) and lysine (L) in bold indicate basic amino acids in HA cleavage site.

Nucleotide sequence comparison of HA and NA between the initial isolate (CPA1) and the other H7N3 viruses in clusters showed a level of similarity ranging from 94.94 percent to 97.87 percent, which was lower than what found in the other segments (PB2, PB1, PA, NP, MP and NS) which ranged from 95.99 to 98.78 percent. In particular, the HA gene exhibited the lowest level of similarity (cluster A: 94.94–96.55%, B1: 95.95–96.07%, and B2: 95.18–96.55%) with CPA1 when compared with other segments (S2 Table).

The multi-basic cleavage site which determines high pathogenicity in poultry has been maintained through all three clusters [3]. The mutations in the HA cleavage site resulted in seven different types of cleavage sites (Table 1). There was no addition or deletions of amino acids in the cleavage site but there are variations in basic amino acids (Arginine and Lysine). Most of the cleavage sites consist of six basic amino acids at or near the cleavage site. Two transitional viruses and one virus in cluster A1 had five muti-basic amino acids, and one virus in cluster A1 had seven. Although there was some variation within clusters, cluster A1 and A2 had one amino acid change, and cluster B had three amino acid changes when compared to the CPA1 virus.

### Estimation of the time of the most recent common ancestor

The coalescent analysis indicates that initial Mexican H7N3 virus was closely related to wild bird-origin viruses as showed in other studies [11, 31]. The time-scaled maximum clade credibility (MCC) trees based on uncorrelated lognormal clock were used to estimate the TMRCA of the Mexican H7N3 viruses (S2 Fig). The TMCRA was estimated to be in April to September 2011 for PB1, PA, HA, NP, and NA gene (Table 2). The estimation of TMRCA for PB2 and NS gene were in September 2010. As the wild bird M gene sequences most related to the initial H7N3 HPAI virus were from 2008, the TMCRA was estimated to be in October 2008 for the M gene.

**Table 2:** TMRCA estimation, nucleotide substitution rate, and dN/dS ratio between the Mexican H7N3 HPAI viruses and related H7 AI wild bird viruses.

	TMRCA [95%HPD]	Posterior probability	Nucleotide substitution rate (10^−3^ substitution/site/year [95% HPD])			dN/dS ratio	
			Global rate by ML ^a^	RLC Wild bird rates ^b^	RLC Poultry rates	Wild birds	Poultry
**PB2**	Sep 25, 2010 [Mar 9, 2010 –Apr 18, 2011]	0.997	2.6	2.6 [2.0–3.2]	6.2 [5.2–7.3]	0.055	0.129
**PB1**	Aug 31, 2011 [Mar 13, 2011 –Feb 8, 2012]	0.995	3.2	2.3 [1.7–3.1]	8.5 [7.0–9.9]	0.041	0.099
**PB1-F2**						5.97	2.97
**PA**	Jun 23, 2011 [Jan 20, 2011 –Nov 24, 2011]	1.000	3.3	2.1 [1.6–2.6]	6.1 [4.9–7.2]	0.037	0.134
**HA**	Sep 13, 2011 [Apr 4, 2011 –Feb 11, 2012]	1.000	5.0	4.9 [4.0–5.7]	10.2 [8.6–11.9]	0.144	0.269
**NP**	Apr 15, 2011 [Oct 24, 2010 –Sep 27, 2011]	0.996	3.2	2.5 [1.9–3.1]	5.6 [4.3–6.9]	0.046	0.075
**NA**	Jun 1, 2011 [Jan 12, 2011 –Oct 13, 2011]	1.000	3.4	5.6 [4.2–6.9]	5.7 [4.7–6.8]	0.094	0.259
**M**	Oct 28, 2008 [Jul 27, 2008 –Dec 11, 2008]	1.000	1.5	2.1 [1.1–3.2]	3.3 [1.9–4.7]		
**M1**						0.020	0.154
**M2**						0.261	0.359
**NS**	Sep 5, 2010 [Apr 12, 2009 –Nov 16, 2011]	0.999	1.6	1.4 [0.8–2.0]	4.4 [3.2–5.9]		
**NS1**						0.162	0.320
**NS2**						0.279	0.155

^a^ Root-to-tip regression analysis was used to calculate global substitution rates by using AI sequences isolated in North America from 2000 to 2017

^b^ Coalescent analysis was used to calculate mean substitution rates by using ancestral sequences constructing monophylogenetic group with the Mexican H7N3 viruses

TMRCA; time of the most recent common ancestor; dN/dS ratio, Non-synonymous to synonymous substitutions ratio, RLC; Random local clock.

### Evolutionary substitution rates

**Fig 2:**
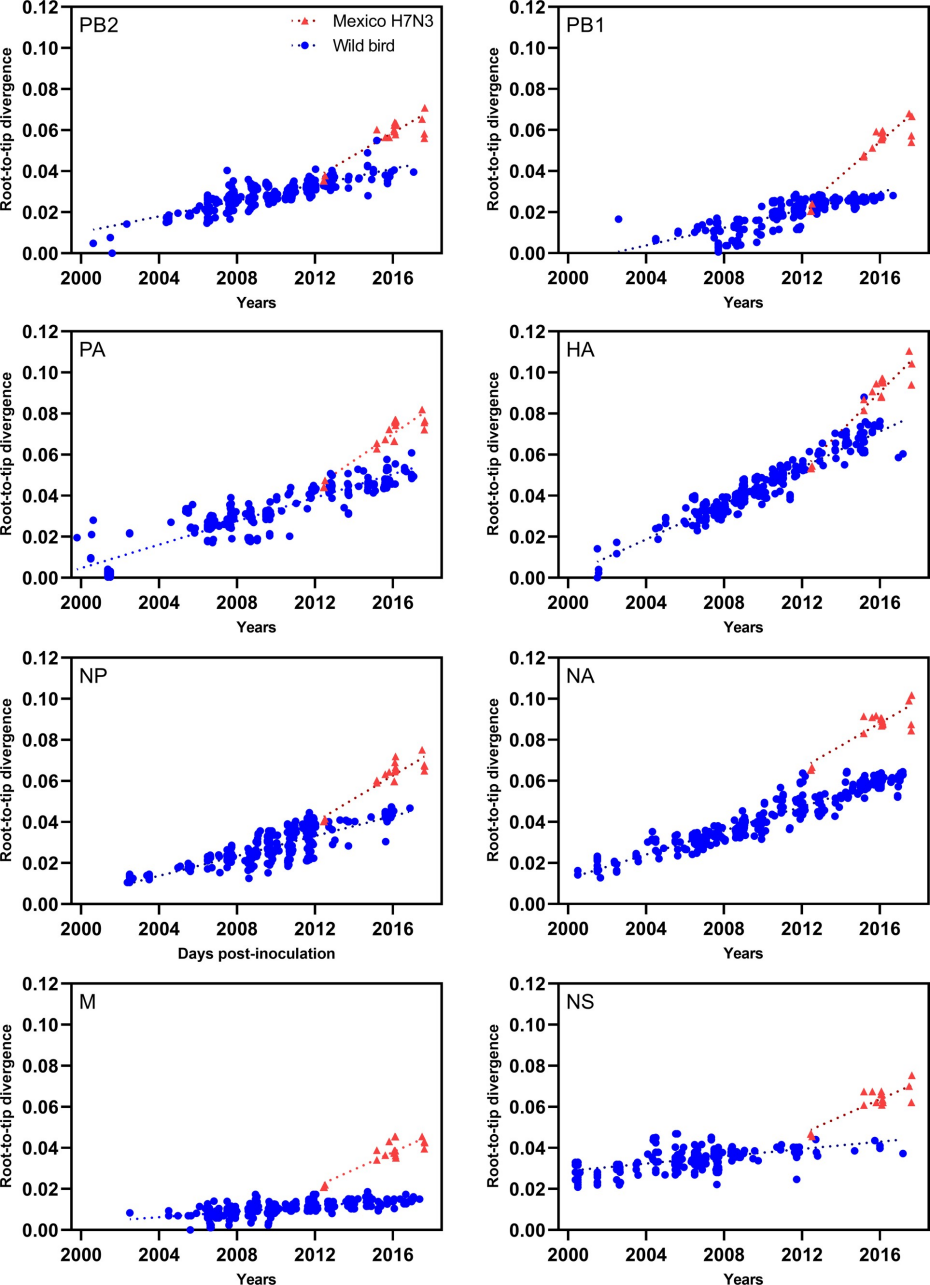
Root-to-tip regression plot of maximum-likelihood phylogeny of each segment of the Mexican H7N3 HPAI viruses. To investigate the temporal signal and clocklikeness of ML phylogenies of the dataset, the linear regression on the root-to-tip distances of samples versus date of the isolate were performed using the Mexican H7N3 HPAI and other North American sequences in 2000–2017. Red triangles and blue circles indicate the Mexican H7N3 HPAI and other North American sequences.

Maximum likelihood phylogenetic trees were generated for the root-to-tip regression analysis using wild bird-origin sequences and the H7N3 Mexico sequences (S4 Fig). Our data showed temporal signal with high correlation coefficient values (PB2: 0.8319, PB1: 0.7765, PA: 0.4389, HA: 0.9327, NP: 0.8232, NA: 0.9111, M: 0.6312, NS: 0.8794), suggesting positive correlations between genetic divergence and sampling time. In general, the global substitution rate of each gene including North American and the H7N3 viruses were in range of global AI virus substitution rates as described elsewhere [32] (Table 2). The root-to-tip regression analysis showed that all Mexican H7N3 sequences were above the estimated regression line (Fig 2). The time-scaled MCC trees based on random local clock were used to estimate the rate before and after an introduction to the Mexican poultry (S3 Fig). The coalescent analysis also showed that the mean substitution rates of wild bird-origin viruses were similar to the rates calculated based on ML and root-to-tip regression analysis (Table 2). However, the NA segment of the wild bird-origin viruses (5.6 × 10^−3^ substitution/site/year) showed a higher mean substitution rate than that of global North American AI viruses (3.4 × 10^−3^ substitution/site/year) calculated by root-to-tip regression analysis. When we compared the mean substitution rate between the wild bird and poultry viruses, the substitution rate of PB2, PB1, PA, HA, NP, M and NS genes were significantly higher in the poultry viruses (Table 2). The fastest evolution was found in the HA (1.02 × 10^−2^ substitution/site/year) while the PB1 gene showed the highest fold increase in rate (2.3 to 8.5 × 10^−3^ substitution/site/year, about 3.7 fold increase). The NA was the only segment that showed no significant rate change.

**Fig 3:**
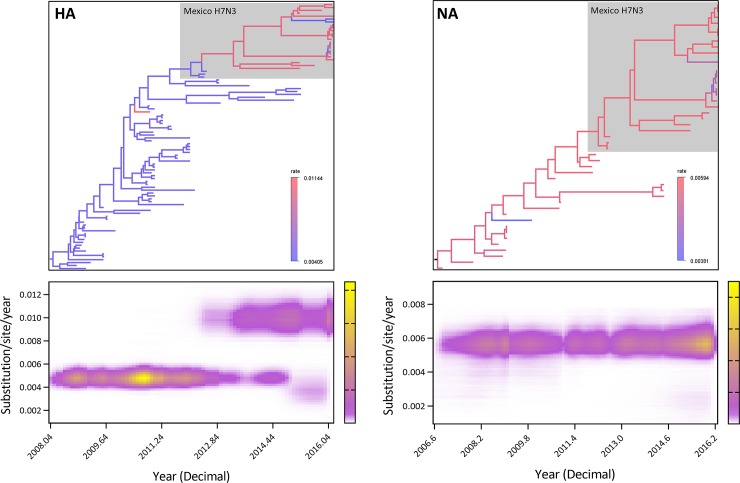
Shift in HA and NA nucleotide substitution rate of the Mexican H7N3 HPAI and related ancestral wild bird viruses. Time-scaled phylogenetic tree of the Mexican H7N3 HPAI viruses and relating ancestral wild birds were depicted. Branch coloring denoted in a gradient scale indicates inferred rates of nucleotide substitution. Gray outlining indicates the Mexican H7N3 HPAI viruses. Rate heterogeneity of hemagglutinin sequence evolution over time was visualized underneath the HA and NA phylogenetic trees. The plot traces the marginal distribution of relative substitution rates across time. White indicates low posterior density, and yellow/pink indicates high density. The estimated rates are higher in HA of the Mexican H7N3 HPAI viruses, with a notable rate shift in approximately 2012 and 2013. In contrast, the rates have no significant change in the NA of the H7N3 viruses.

Fig 3A depicts the Bayesian consensus tree of HA, along with posterior mean branch lengths scaled in real time. To examine rate variation, we colored branches by mean relative rate of nucleotide substitution in gradient scale, with > 0.5 posterior probability. A radical shift of rate begins after the initial H7N3 HPAI outbreak with increasing rate variation; most branches of wild birds and initial Mexican H7N3 viruses are blue, while the later Mexican H7N3 branches appear mostly red except two viruses from 2017 in cluster A1 and some closely related viruses in cluster B. Fig 3B compares the posterior and prior mass functions relating the number of rate changes observed in HA evolution. Rates colored yellow had high posterior probability, while rates proceeding towards purple reflect lower probabilities. As expected from the observed variation in Fig 3A, no posterior mass falls on the existence of a global clock without rate changes; the median modal number of the rate change is three. The lower 95% HPD value of the coefficient of variation was 0.327, indicative of rate change among the lineage. Excluding the rate change of an internal branch of two outliers in 2017 (A/chicken/Jalisco/7DIEGO/2017 and A/chicken/7LG/2017) and the short internal branches in cluster B, the one major rate break points in Fig 3B generated two distinct rates in HA evolution.

### Comparison of dN/dS and transition-to-transversion ratio between wild birds and poultry H7N3 virus sequences

The dN/dS ratio was calculated to investigate whether the H7N3 introduction into poultry impacted amino acid change (Table 2). An increase in dN/dS ratios were observed in all of the viral proteins except PB1-F2 and NS2. To identify which type of nucleotide substitution was favored in the amino acid changes, the transition-to-transversion ratios were calculated in wild bird and poultry sequences. The transition-to-transversion ratio of the PB2, PB1, PA, HA, M2 and NS2 genes decreased after introduction of the virus into poultry, whereas PB1-F2, NP, NA, M1 and NS1 genes showed no or increased transition-to-transversion ratio (S5 Table).

### Detection of selective pressure and prediction of N-glycosylation in HA and NA

The selective pressures were estimated using five methods implemented in Datamonkey and BEAST program. When estimated with SLAC, the global dN/dS ratios for all gene segments were lower than one, except for PB1-F2, which shows that strong purifying selection acted on the evolution of the virus. However, some amino acids were subject to positive selection. Forty-nine positively selected sites were detected by one or more methods in the Mexican H7N3 lineage (Table 3). Among those positively selected sites, 18 sites were detected by more than one method (position 116, 389 in PB2; position 216 in PB1; position 396, 668 in PA; position 52, 73, 130, 133, 157, 201 in HA; positions 249, 397, 412 in NA; positions 23 in M2; position 112, 171, 180 in NS1). No positive selection was identified in NP and M1. All the amino acid sequences at the 18 positively selected sites in the Mexican H7N3 HPAI viruses were more frequently found in poultry than in wild birds available in GenBank (S6 Table). Some amino acid changes in the PB2 (K116R), HA (D73K/N/S and R130T), NA (N249S, V397M and K412R) and NS1 (A112T, D171G/N, V180T) were found in higher proportion in poultry.

**Table 3:** Summary of positive selection sites in the proteins of the Mexican H7N3 HPAI viruses.

	Method of positive selection ^a^					ω > 1 (total no. of sites) ^b^	Positively selected sites in H7N3 ^c^
	SLAC	FEL	MEME	FUBAR	Renaissance^d^		
PB2	-	389	88, 109, 116, 355, 676	116, 389	116, 389	6	K116R, R389K
PB1	-	215, 216	-	216	216	2	S216I
PB1-F2	-	-	-	-	-	-	-
PA	-	108, 215, 358, 753	85, 396	350, 396, 668	350, 396, 554, 595, 668	11	D396Y, I668V
HA(H7 numbering)	130	52, 73, 86, 130, 133, 157, 201, 518	130, 133, 157, 201, 361, 537	130, 157, 271	49, 52, 73, 130, 133, 146, 148, 157, 205, 258, 267, 271, 392, 412, 461, 518	6	G52K/E/R, D73K/N, R130K/T, G133S/N, K157Q/M/R, Q201L
NP	-	-	-	-	-	-	-
NA	-	389, 397	81, 140, 357	249, 412	45, 191, 249, 397, 412	9	N249S, V397M, K412R
M1	-	-	-	-	-	-	-
M2	-	-	-	23	23	1	S23N
NS1	-	-	180	112, 171	60, 67, 112, 139, 171, 180, 191, 209, 216. 224	10	A112T, D171G/N, V180T
NS2	-	-	-	-	3, 14, 27, 52	4	-

^a^ Among the total number of positively selected sites, amino acid sites with statistically significant levels (p ≤ 0.1 (SLAC, FEL, and MEME) posterior probability ≥ 0.9 (FUBAR)) are indicated.

^b^ The total number of positively selected sites (ω > 1) in are counted from different methods in Mexican H7N3 viruses.

^c^ Positive sites confirmed by more than one method are indicated. The amino acids set in before and after a site number represent amino acid changes found in wild bird-origin viruses (before) to the Mexican H7N3 viruses (after).

^d^ Renaissance counting implemented in BEAST.

The prediction of N-linked glycosylation sites in the HA protein sequences revealed that the Mexican H7N3 lineage had nine potential glycosylation sites in the HA1-coding region (position 12, 28, 123, 133, 149, 154, 164, 205, and 231 using H7 numbering) with a range of 3–7 sites for individual isolates (S3 Table). Initially, the index H7N3 virus (CPA1) was found to have three potenital glycosylation sites in position 12, 46, and 231. Then three 2015 isolates gained three additional glycosylation site in position 123, 133, and 149. The three acquitition have been maintained in most isolates of the cluster A with one additional site in position 164. On the other had, cluster B acquired only two additional glycosylation site in position 123 and 149 compared to the CPA1. In particular, one isolate (A/chicken/Jalisco/716/2017)in cluster A1 acquired a potential glycosylation at position 205 but it lost a potential glycosylation at 123. Likewise, while one isolate (A/chicken/Jalisco/PAVX17170/2017) in cluster B acquired a potential glycosylation site at position 154, it lost a glycosylation in 123.

Eight potential glycosylation sites in the NA coding region were predicted at position 14, 57, 66, 72, 146, 308, 341 and 457 with a range of 6–8 sites for individual isolates (S4 Table). The 66, 72, 146 and 308 potential glycosylation sites in NA were maintained in all Mexican H7N3. The NA sequences in cluster B potentially lost a glycosylation in position 14. The two 2015 isolates lsot potential glycosylation in position 57. One virus in the cluster A (A/chicken/Guanajuato/07437_15/2015) resulted potential gain of glycosylation in position 341.

## Discussion

The estimation of TMRCA demonstrated that the eight genes of the first Mexican H7N3 AI virus most likely emerged in 2011. Once the virus transmitted to poultry, it acquired multiple basic amino acid in the HA cleavage site through recombination with chicken 28S rRNA changing into the highly pathogenic form [9]. Our results show the H7N3 HPAI virus has diverged into at least three genetic clusters with different genetic mutations in HA cleavage site sequences. Most of the viruses evolved with no reassortment between clusters, maintaining their geographical distribution. We also found that there was no reassortment with H5N2 LPAI virus which has been endemic in Mexico since 1994 [33]. However, three viruses had evidence of reassortment within the H7N3 lineage, with heterologous PB2, PB1 and NS genes, indicating that the the different viral clusters had some overlap in their range. In addition, considering the geological proximity to the Chiapas region, it appears that the outbreak in wild birds in 2015 (in *Ortalis vetula*, *Turdus grayi and Amazon albifrons*) is associated with spillover of virus from poultry with the closest identity in Oaxaca [34].

By comparing the sequences of the wild bird AI viruses and the Mexican H7N3 HPAI poultry viruses we found that the dN/dS ratios in PB2, PB1, PA, HA, NP, NA, M1, M2 and NS1 increased after introduction of the virus into poultry, suggesting that the H7N3 virus has evolved under higher selective pressure in poultry than in wild birds possibly due to host adaptation and/or pre-existing immunity against AI virus. The decreased transition-to-transversion ratios in PB2, PB1, PA, HA, M2 and NS2 also confirmed that the proportion of transversion substitutions increased. However, the similar or increased transition-to-transversion ratio of the PB1-F2, NP, NA, M1 and NS1 genes after the introduction shows that the amino acid changes are dominated by non-synonymous transition substitutions, which are more likely to conserve biochemical properties of the proteins [35, 36].

The Mexican H7N3 HPAI outbreak in poultry is the second longest H7 HPAI outbreak, following the Pakistani H7N3 HPAI outbreak (1995–2004) [37]. While the source of the Pakistani outbreak remained unknown due to limited virus genetic information, active AI surveillance in North American wild birds provided a large pool for sequencing and analysis which enabled the identification of the likely precursor of the Mexican H7N3 HPAI virus causing the outbreak. Both the Mexico and the Pakistani outbreaks were counteracted by vaccination using an inactivated wild bird H7N3 strains, A/cinnamon teal/Mexico/2817/2006 for Mexico, and an autologous poultry isolate for Pakistan, which conferred protective immunity against the earlier outbreak viruses [10, 38, 39]. The Pakistani H7N3 HPAI virus did not show significant evolution, with no detectable selection of antigenic-drift variants in HA [38], whereas our results show that the Mexican H7N3 underwent rapid evolution compared to its wild bird ancestors. Similar high substitution rates were also observed in an Italian H7N1 outbreak, where the HPAI virus emerged from its low pathogenic form and vaccination was implemented by using a low pathogenic AI strain from chicken [40, 41]. However, the mean substitution rate of the PB1 gene of the Mexican H7N3 (8.5 × 10^−3^ substitution/site/year) was higher than that of the Italian H7N1 (5.5–5.7 × 10^−3^ substitution/site/year). In contrast, the mean substitution rates of the NA, M, and NS of the Mexican H7N3 were not as high as the rates observed in the Italian H7N1. These data suggest that the changes in evolutionary rate, which might be driven by selection and adaptation of the AI virus to a new host or production system, can be different for each virus strain and can vary at the segment level.

The increased substitution rate and amino acid changes that are positively selected in the Mexican H7N3 viruses could be explained in three ways. First, there is a higher opportunity for the virus to infect and replicate in poultry than in wild birds. High host density and frequent contact in poultry setting may lead to exposure to greater quantity of susceptible hosts and higher virus replication, and hence a greater number of mutations per unit time [42]. In Mexico, the layer farms delivering over 70% of the eggs of the entire production of supply for Mexico are centralized in the Jalisco, Puebla and Guanajuato regions where the H7N3 outbreaks were reported [43] giving the virus ample opportunity to spread and replicate in millions of individual poultry. In contrast, the latent period associated with environmental transmission may reduce substitution rates in wild birds [44, 45]. Second, as the H7N3 virus circulated in poultry for several years, amino acid changes related to poultry adaptation were likely to occur at the positively selected sites. For example, mutation in position 130, which is located in the receptor binding region of HA, was also positively selected in association with host shift in evolution of AI H7 virus analysis [46]. The mutations in position 157 and 201 locate in the receptor binding region, and the mutation in position 52 and 73 are possibly associated with membrane fusion or pH stability [47]. Other amino acid changes in higher proportion in North American poultry sequences compared to wild bird virus sequences are possibly associated with adaptation of the Mexican H7N3 HPAI viruses in chickens (S6 Table); PB2 (K116R), HA (D73K/N/S and R130T), NA (N249S, V397M and K412R), and NS1 (A112T, D171G/N, V180T). Third, the immune status of the poultry could have also increased the selective pressure on the virus. Vaccination was widely instituted in Mexico using the LPAI virus A/Cinnamon Teal/Mexico/2817/2006(H7N3) which experimentally was shown to be protective from challenge with the virulent virus. The nucleotide sequence relatedness between this wild bird origin vaccine strain and the field HPAI strains varied from 90.5% to 98.1% for all gene segments, and likely provided opportunities for greater virus replication and opportunities for positive selection [10]. This vaccine although protective early on, appeared to have reduced effectiveness after just a few years. Although a direct association between H7N3 vaccination and virus evolution has not been fully established, there is evidence that addition of N-glycosylation sites contributed to the escape of 2015 Mexican H7N3 HPAI from vaccine-induced immunity [16]. In addition, considering that the AI virus evolves more rapidly in domestic poultry than in wild birds, the high evolution rate and dn/ds ratio of HA in the Mexican poultry viruses, which is comparable to the rate of Italian H7N1 and Indonesian H5N1 outbreaks, may also imply selective pressure caused by vaccine-induced immunity [40, 48].

Since the HA and NA of the early vaccine strain (A/cinnamon teal/Mexico/2817/2006) has the same sequences to the wild bird viruses close to the Mexican H7N3 viruses, all the positively selected sites in HA and NA correspond to changes in the vaccine strain. However, some amino acid changes found in the Mexican H7N3 viruses correspond to other mutations in relation to vaccines. The 123 glycosylation in HA was also found in Pakistani, Dutch, and Italian H7 HPAI viruses, however vaccination was not used during the Dutch and Italian outbreaks [38, 40, 49]. Similar amino acid changes can be found in H5 homologs. Although it is not an exact match, gain of potential glycosylation at position 123 was also observed in the Mexican lineage H5N2 escape mutants at position 126 (position 121 in H7 numbering) [50]. The G133S/N mutation in the H7N3 HA, where positive selection caused potential N-glycosylation site in antigenic site A, also corresponds to the mutation that contributed to the antigenic drift in the Egyptian H5N1 HPAI virus [51]. The R130K/T mutation in HA is also present in H5 vaccine escape mutants at position 136 [50, 52, 53]. Between the two positively selected sites in HA, the G133S/N mutation was located in an experimentally proven epitope region [54].

This study is the first analysis demonstrating the evolution of the Mexican H7N3 HPAI after five years of circulation in poultry. After introduction of the Mexican H7N3 HPAI virus to poultry the viral genome has changed greatly above the normal global substituion rate, with a strong negative selection. As a consequence, the virus has diversified into three clusters with genetic variations in HA cleavage sites. Amino acids changes that are positively selected in the H7N3 virus suggest adaptation to poultry and possible vaccine escape mutations as mechanisms of change during the virus evolution. Continuous monitoring and genetic analysis are needed to better understand the evolution of HPAI viruses persisting in poultry and to control the virus spread.

## Supporting information

S1 Fig Maximum likelihood trees of the H7N3 HPAI. (PDF)

S2 Fig Time-scaled phylogenetic trees of the H7N3 HPAI and related wild bird viruses with bars to each node to display 95% HPD of the height. (PDF)

S3 Fig Time-scaled phylogenetic trees of the H7N3 HPAI and related wild bird viruses with branches colored by the nucleotide substitution rates. (PDF)

S4 Fig Maximum likelihood phylogenetic trees used for the root-to-tip regression analysis. Closed circles are viruses isolated and sequenced in this study. Brackets indicate genetic cluster of the H7N3 HPAI.
(PDF)

S1 Table The H7N3 HPAI viruses isolated in this study (strain, collection date and Genbank accession number). (DOCX)

S2 Table Comparison of nucleotide similarity in percentage between the initial isolate (CPA1) and subclusters. (DOCX)

S3 Table N-glycosylation site prediction in HA proteins. (DOCX)

S4 Table N-glycosylation site prediction in the NA proteins. (DOCX)

S5 Table Maximum likelihood estimate of transition-to-transversion ratio. (DOCX)

S6 Table Amino acid distribution of the positively selected sites in the North American H7 HA protein. (DOCX)
