# Supplementary material for: Rapid evolution of Mexican H7N3 highly pathogenic avian influenza viruses in poultry
Source: PLoS One. 2019 Sep 12;14(9):e0222457. doi: 10.1371/journal.pone.0222457 (PMC6742402; doi:10.1371/journal.pone.0222457)
Supplement: S1 Fig — (PDF) [file pone.0222457.s001.pdf]

Supplementary Figures 1 – Maximum likelihood trees (The closed circles indicate viruses isolated in this study)

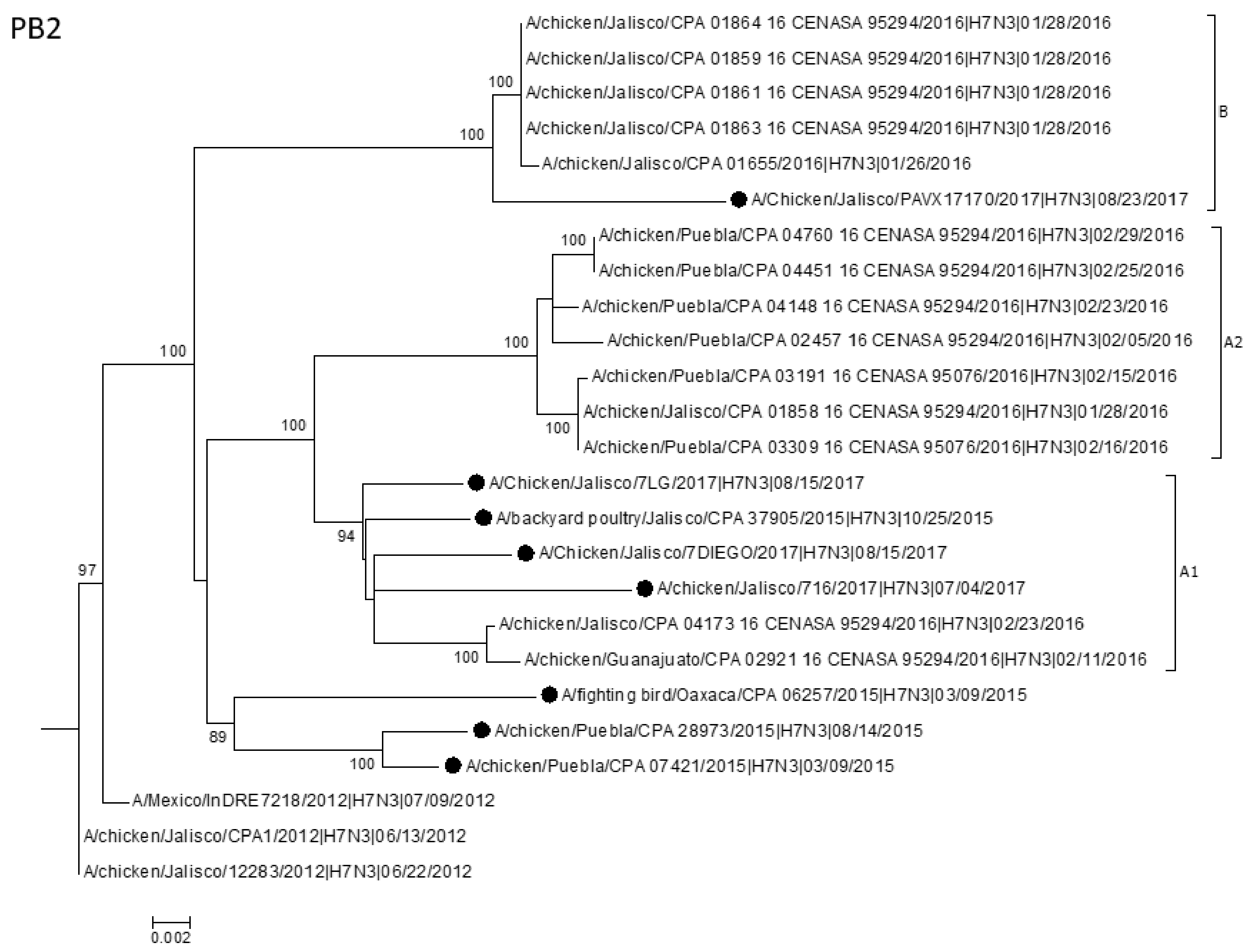

PB1

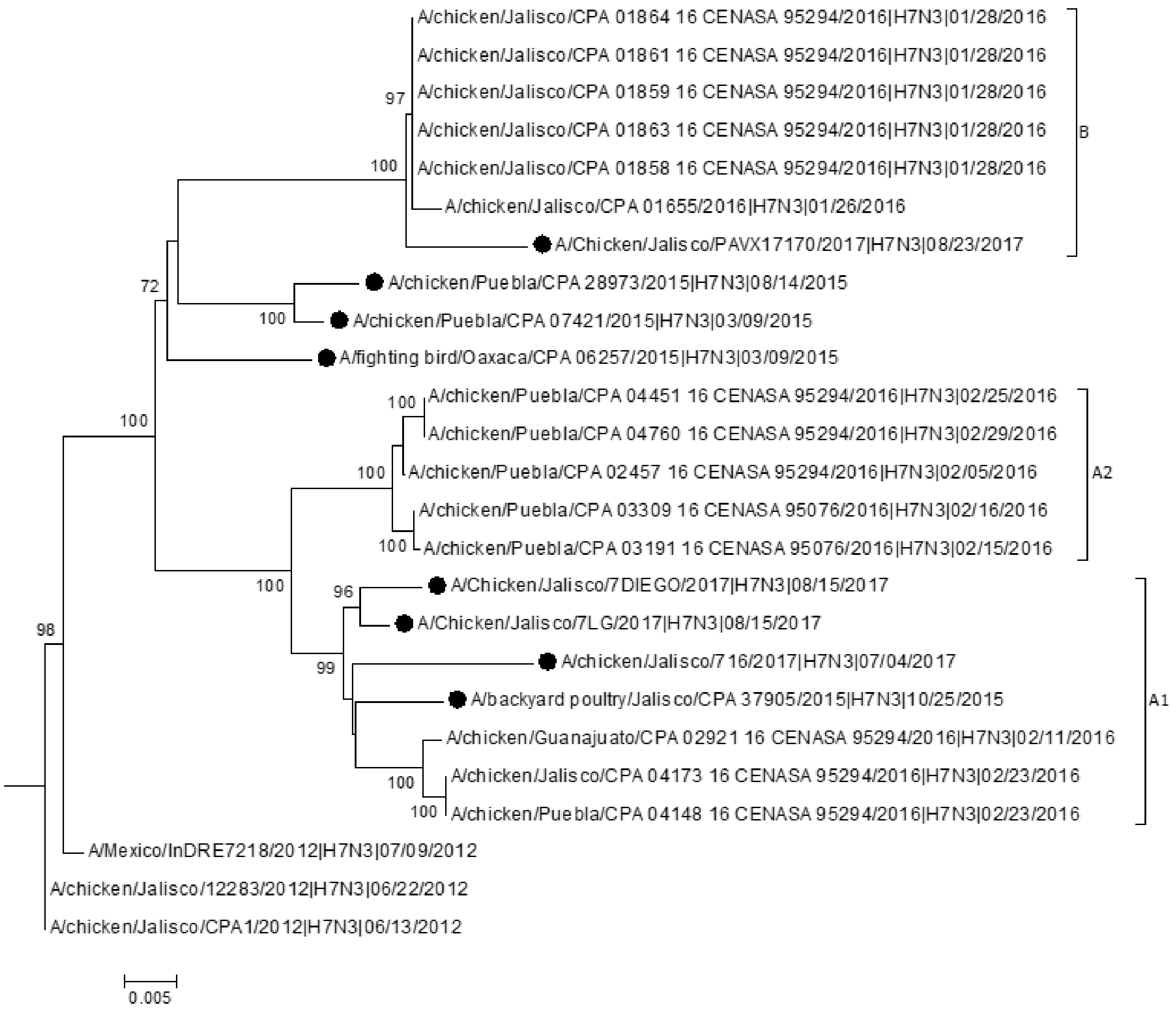

A/chicken/Jalisco/CPA 01864 16 CENASA 95294/2016|H7N3|01/28/2016
A/chicken/Jalisco/CPA 01861 16 CENASA 95294/2016|H7N3|01/28/2016
A/chicken/Jalisco/CPA 01859 16 CENASA 95294/2016|H7N3|01/28/2016
A/chicken/Jalisco/CPA 01863 16 CENASA 95294/2016|H7N3|01/28/2016
A/chicken/Jalisco/CPA 01858 16 CENASA 95294/2016|H7N3|01/28/2016
A/chicken/Jalisco/CPA 01655/2016|H7N3|01/26/2016
A/Chicken/Jalisco/PAVX17170/2017|H7N3|08/23/2017
B
A/chicken/Puebla/CPA 28973/2015|H7N3|08/14/2015
A/chicken/Puebla/CPA 07421/2015|H7N3|03/09/2015
A/fighting bird/Oaxaca/CPA 06257/2015|H7N3|03/09/2015
A/chicken/Puebla/CPA 04451 16 CENASA 95294/2016|H7N3|02/25/2016
A/chicken/Puebla/CPA 04760 16 CENASA 95294/2016|H7N3|02/29/2016
A/chicken/Puebla/CPA 02457 16 CENASA 95294/2016|H7N3|02/05/2016
A/chicken/Puebla/CPA 03309 16 CENASA 95076/2016|H7N3|02/16/2016
A/chicken/Puebla/CPA 03191 16 CENASA 95076/2016|H7N3|02/15/2016
A2
A/Chicken/Jalisco/7DIEGO/2017|H7N3|08/15/2017
A/Chicken/Jalisco/7LG/2017|H7N3|08/15/2017
A/chicken/Jalisco/716/2017|H7N3|07/04/2017
A/backyard poultry/Jalisco/CPA 37905/2015|H7N3|10/25/2015
A/chicken/Guanajuato/CPA 02921 16 CENASA 95294/2016|H7N3|02/11/2016
A/chicken/Jalisco/CPA 04173 16 CENASA 95294/2016|H7N3|02/23/2016
A/chicken/Puebla/CPA 04148 16 CENASA 95294/2016|H7N3|02/23/2016
A1
A/Mexico/InDRE7218/2012|H7N3|07/09/2012
A/chicken/Jalisco/12283/2012|H7N3|06/22/2012
A/chicken/Jalisco/CPA1/2012|H7N3|06/13/2012
97
100
72
100
100
100
100
100
100
96
99
98
100
100
0.005

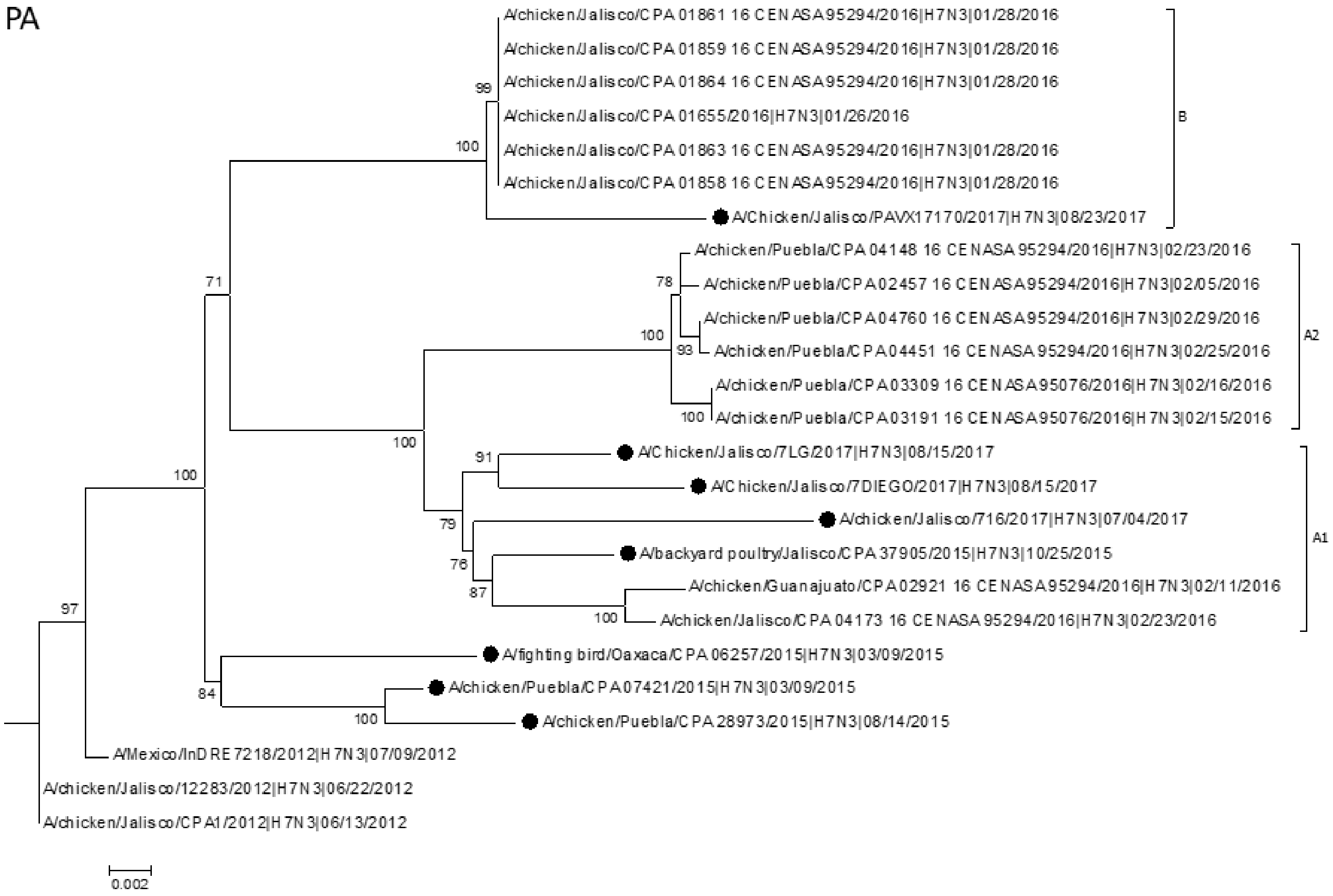
PA
A/chicken/Jalisco/CPA 01861 16 CENASA95294/2016|H7N3|01/28/2016
A/chicken/Jalisco/CPA 01859 16 CENASA95294/2016|H7N3|01/28/2016
A/chicken/Jalisco/CPA 01864 16 CENASA95294/2016|H7N3|01/28/2016
A/chicken/Jalisco/CPA 01655/2016|H7N3|01/26/2016
A/chicken/Jalisco/CPA 01863 16 CENASA95294/2016|H7N3|01/28/2016
A/chicken/Jalisco/CPA 01858 16 CENASA95294/2016|H7N3|01/28/2016
A/Chicken/Jalisco/PAVX17170/2017|H7N3|08/23/2017
B
A/chicken/Puebla/CPA 04148 16 CENASA 95294/2016|H7N3|02/23/2016
A/chicken/Puebla/CPA 02457 16 CENASA95294/2016|H7N3|02/05/2016
A/chicken/Puebla/CPA 04760 16 CENASA95294/2016|H7N3|02/29/2016
A/chicken/Puebla/CPA 04451 16 CENASA 95294/2016|H7N3|02/25/2016
A/chicken/Puebla/CPA 03309 16 CENASA95076/2016|H7N3|02/16/2016
A/chicken/Puebla/CPA 03191 16 CENASA95076/2016|H7N3|02/15/2016
A2
A/Chicken/Jalisco/7LG/2017|H7N3|08/15/2017
A/Chicken/Jalisco/7DIEGO/2017|H7N3|08/15/2017
A/chicken/Jalisco/716/2017|H7N3|07/04/2017
A/backyard poultry/Jalisco/CPA 37905/2015|H7N3|10/25/2015
A/chicken/Guanajuato/CPA 02921 16 CENASA95294/2016|H7N3|02/11/2016
A/chicken/Jalisco/CPA 04173 16 CENASA 95294/2016|H7N3|02/23/2016
A1
A/fighting bird/Oaxaca/CPA 06257/2015|H7N3|03/09/2015
A/chicken/Puebla/CPA 07421/2015|H7N3|03/09/2015
A/chicken/Puebla/CPA 28973/2015|H7N3|08/14/2015
A/Mexico/InDRE7218/2012|H7N3|07/09/2012
A/chicken/Jalisco/12283/2012|H7N3|06/22/2012
A/chicken/Jalisco/CPA1/2012|H7N3|06/13/2012
99
100
71
78
100
93
100
100
91
79
76
87
100
97
84
100
100
0.002

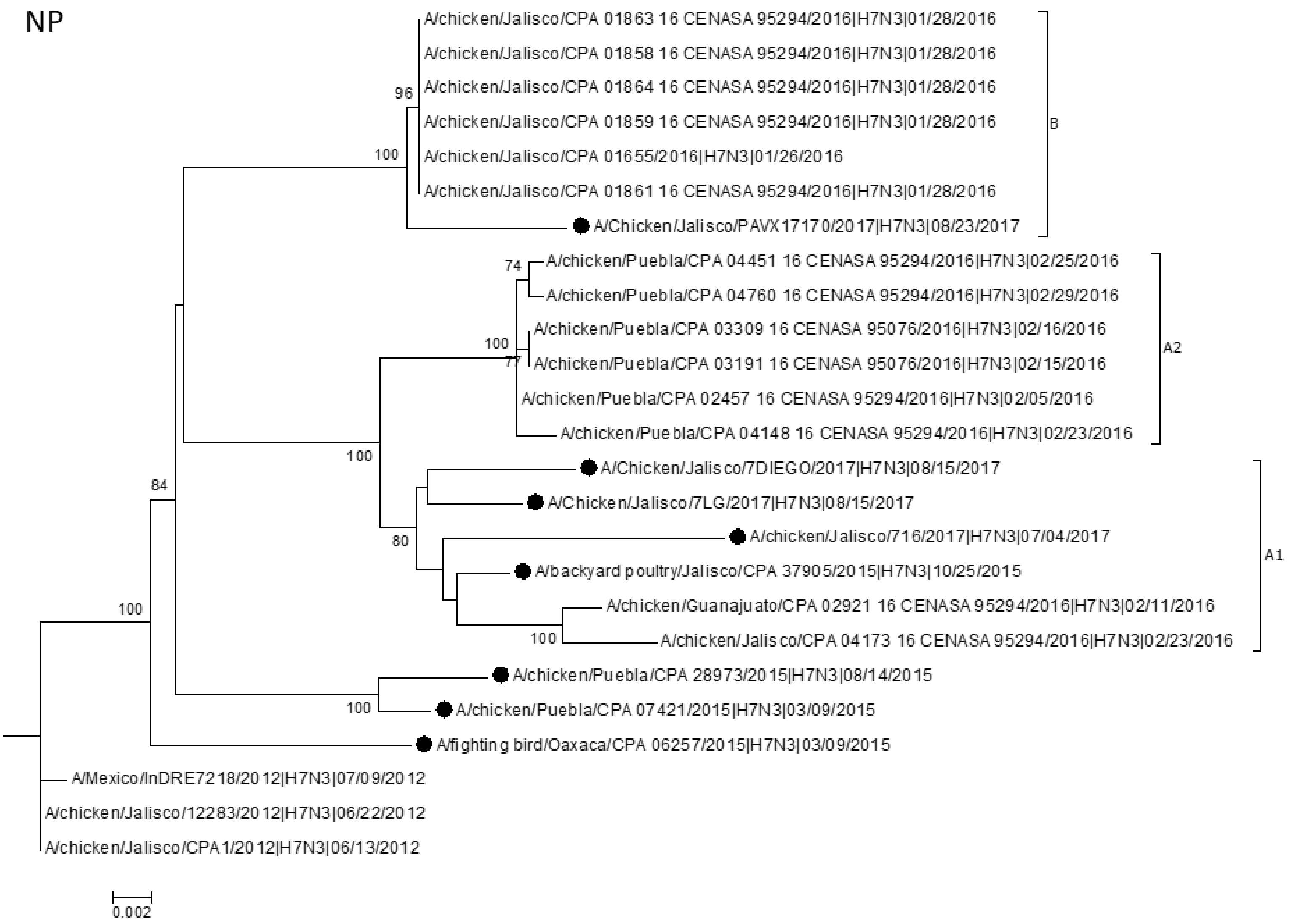
NP
A/chicken/Jalisco/CPA 01863 16 CENASA 95294/2016|H7N3|01/28/2016
A/chicken/Jalisco/CPA 01858 16 CENASA 95294/2016|H7N3|01/28/2016
A/chicken/Jalisco/CPA 01864 16 CENASA 95294/2016|H7N3|01/28/2016
A/chicken/Jalisco/CPA 01859 16 CENASA 95294/2016|H7N3|01/28/2016
A/chicken/Jalisco/CPA 01655/2016|H7N3|01/26/2016
A/chicken/Jalisco/CPA 01861 16 CENASA 95294/2016|H7N3|01/28/2016
A/Chicken/Jalisco/PAVX17170/2017|H7N3|08/23/2017
B
A/chicken/Puebla/CPA 04451 16 CENASA 95294/2016|H7N3|02/25/2016
A/chicken/Puebla/CPA 04760 16 CENASA 95294/2016|H7N3|02/29/2016
A/chicken/Puebla/CPA 03309 16 CENASA 95076/2016|H7N3|02/16/2016
A/chicken/Puebla/CPA 03191 16 CENASA 95076/2016|H7N3|02/15/2016
A/chicken/Puebla/CPA 02457 16 CENASA 95294/2016|H7N3|02/05/2016
A/chicken/Puebla/CPA 04148 16 CENASA 95294/2016|H7N3|02/23/2016
A2
A/Chicken/Jalisco/7DIEGO/2017|H7N3|08/15/2017
A/Chicken/Jalisco/7LG/2017|H7N3|08/15/2017
A/chicken/Jalisco/716/2017|H7N3|07/04/2017
A/backyard poultry/Jalisco/CPA 37905/2015|H7N3|10/25/2015
A/chicken/Guanajuato/CPA 02921 16 CENASA 95294/2016|H7N3|02/11/2016
A/chicken/Jalisco/CPA 04173 16 CENASA 95294/2016|H7N3|02/23/2016
A1
A/chicken/Puebla/CPA 28973/2015|H7N3|08/14/2015
A/chicken/Puebla/CPA 07421/2015|H7N3|03/09/2015
A/fighting bird/Oaxaca/CPA 06257/2015|H7N3|03/09/2015
A/Mexico/InDRE7218/2012|H7N3|07/09/2012
A/chicken/Jalisco/12283/2012|H7N3|06/22/2012
A/chicken/Jalisco/CPA1/2012|H7N3|06/13/2012
96
100
74
100
77
100
80
100
84
100
100
0.002

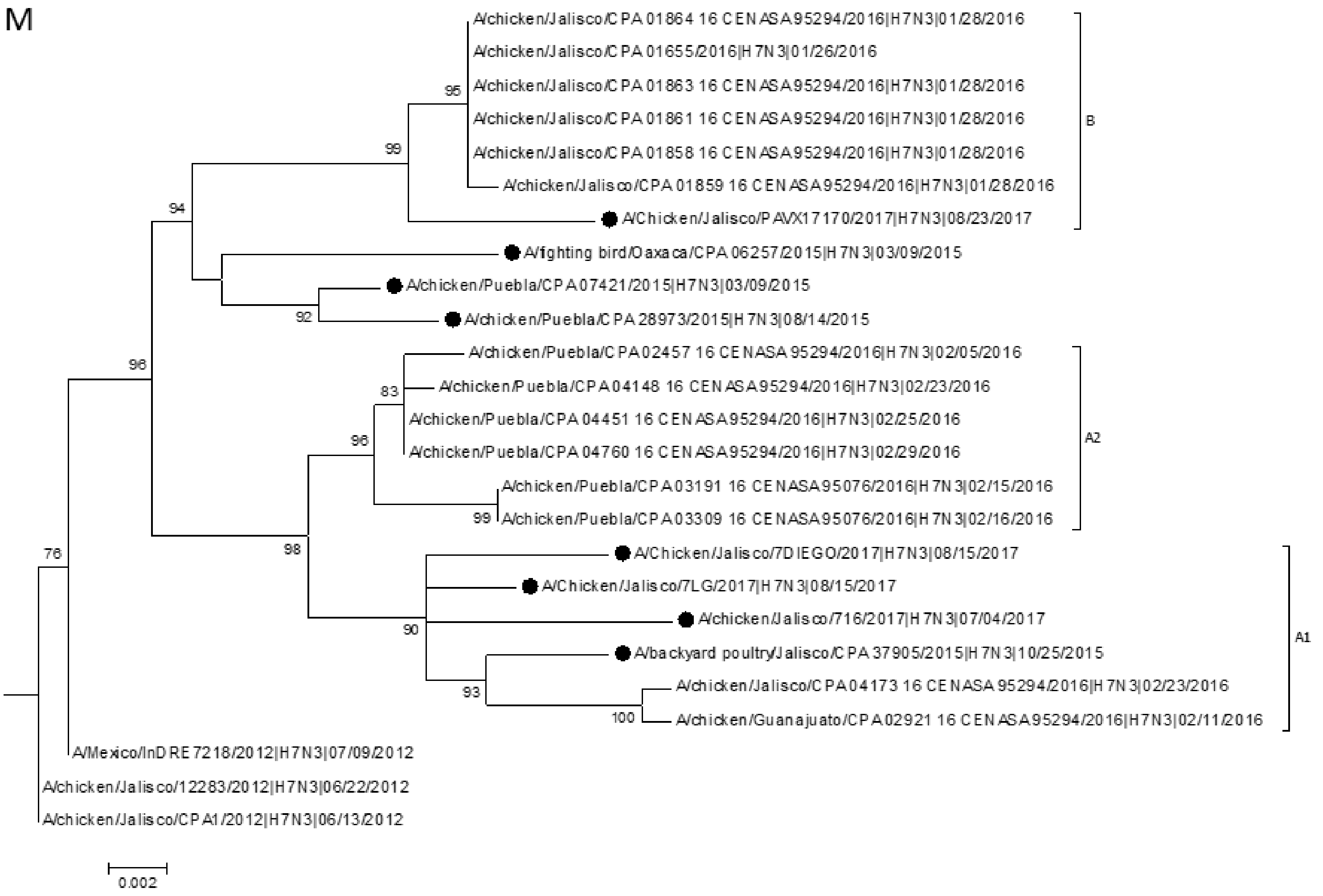
M
A/chicken/Jalisco/CPA 01864 16 CENASA95294/2016|H7N3|01/28/2016
A/chicken/Jalisco/CPA 01655/2016|H7N3|01/26/2016
A/chicken/Jalisco/CPA 01863 16 CENASA95294/2016|H7N3|01/28/2016
A/chicken/Jalisco/CPA 01861 16 CENASA95294/2016|H7N3|01/28/2016
A/chicken/Jalisco/CPA 01858 16 CENASA95294/2016|H7N3|01/28/2016
A/chicken/Jalisco/CPA 01859 16 CENASA95294/2016|H7N3|01/28/2016
A/Chicken/Jalisco/PAVX17170/2017|H7N3|08/23/2017
A/fighting bird/Oaxaca/CPA 06257/2015|H7N3|03/09/2015
A/chicken/Puebla/CPA 07421/2015|H7N3|03/09/2015
A/chicken/Puebla/CPA 28973/2015|H7N3|08/14/2015
A/chicken/Puebla/CPA 02457 16 CENASA 95294/2016|H7N3|02/05/2016
A/chicken/Puebla/CPA 04148 16 CENASA95294/2016|H7N3|02/23/2016
A/chicken/Puebla/CPA 04451 16 CENASA95294/2016|H7N3|02/25/2016
A/chicken/Puebla/CPA 04760 16 CENASA95294/2016|H7N3|02/29/2016
A/chicken/Puebla/CPA 03191 16 CENASA95076/2016|H7N3|02/15/2016
A/chicken/Puebla/CPA 03309 16 CENASA95076/2016|H7N3|02/16/2016
A/Chicken/Jalisco/7DIEGO/2017|H7N3|08/15/2017
A/Chicken/Jalisco/7LG/2017|H7N3|08/15/2017
A/chicken/Jalisco/716/2017|H7N3|07/04/2017
A/backyard poultry/Jalisco/CPA 37905/2015|H7N3|10/25/2015
A/chicken/Jalisco/CPA 04173 16 CENASA 95294/2016|H7N3|02/23/2016
A/chicken/Guanajuato/CPA 02921 16 CENASA95294/2016|H7N3|02/11/2016
A/Mexico/InDRE7218/2012|H7N3|07/09/2012
A/chicken/Jalisco/12283/2012|H7N3|06/22/2012
A/chicken/Jalisco/CPA1/2012|H7N3|06/13/2012
B
A2
A1
95
99
94
92
96
83
96
99
98
76
90
93
100
0.002

NS

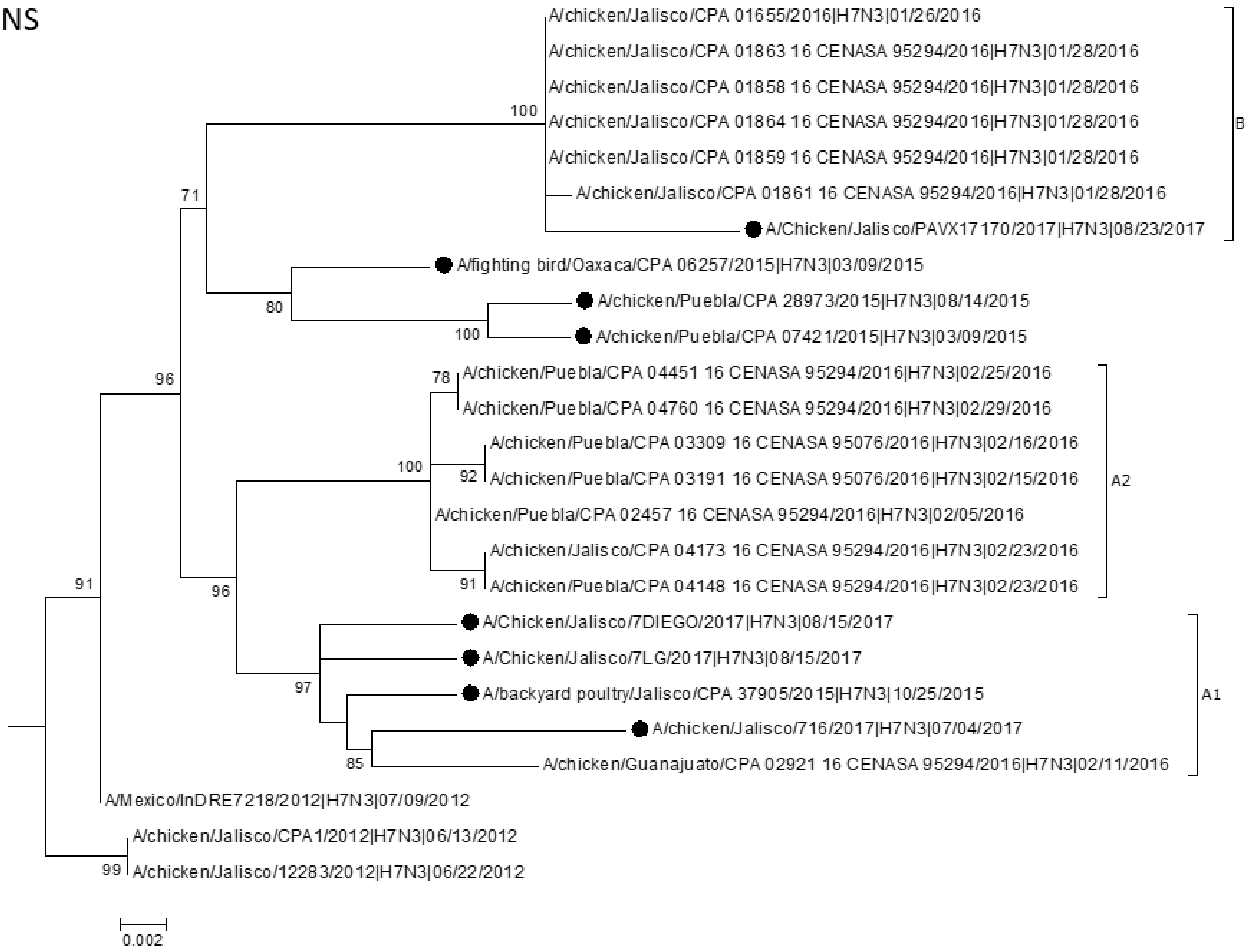

A/chicken/Jalisco/CPA 01655/2016|H7N3|01/26/2016
A/chicken/Jalisco/CPA 01863 16 CENASA 95294/2016|H7N3|01/28/2016
A/chicken/Jalisco/CPA 01858 16 CENASA 95294/2016|H7N3|01/28/2016
A/chicken/Jalisco/CPA 01864 16 CENASA 95294/2016|H7N3|01/28/2016
A/chicken/Jalisco/CPA 01859 16 CENASA 95294/2016|H7N3|01/28/2016
A/chicken/Jalisco/CPA 01861 16 CENASA 95294/2016|H7N3|01/28/2016
A/Chicken/Jalisco/PAVX17170/2017|H7N3|08/23/2017
B
A/fighting bird/Oaxaca/CPA 06257/2015|H7N3|03/09/2015
A/chicken/Puebla/CPA 28973/2015|H7N3|08/14/2015
A/chicken/Puebla/CPA 07421/2015|H7N3|03/09/2015
A/chicken/Puebla/CPA 04451 16 CENASA 95294/2016|H7N3|02/25/2016
A/chicken/Puebla/CPA 04760 16 CENASA 95294/2016|H7N3|02/29/2016
A/chicken/Puebla/CPA 03309 16 CENASA 95076/2016|H7N3|02/16/2016
A/chicken/Puebla/CPA 03191 16 CENASA 95076/2016|H7N3|02/15/2016
A/chicken/Puebla/CPA 02457 16 CENASA 95294/2016|H7N3|02/05/2016
A/chicken/Jalisco/CPA 04173 16 CENASA 95294/2016|H7N3|02/23/2016
A/chicken/Puebla/CPA 04148 16 CENASA 95294/2016|H7N3|02/23/2016
A2
A/Chicken/Jalisco/7DIEGO/2017|H7N3|08/15/2017
A/Chicken/Jalisco/7LG/2017|H7N3|08/15/2017
A/backyard poultry/Jalisco/CPA 37905/2015|H7N3|10/25/2015
A/chicken/Jalisco/716/2017|H7N3|07/04/2017
A/chicken/Guanajuato/CPA 02921 16 CENASA 95294/2016|H7N3|02/11/2016
A1
A/Mexico/InDRE7218/2012|H7N3|07/09/2012
A/chicken/Jalisco/CPA1/2012|H7N3|06/13/2012
A/chicken/Jalisco/12283/2012|H7N3|06/22/2012
100
71
80
100
96
78
100
92
91
96
91
97
85
99
0.002
