# Supplementary material for: Rapid evolution of Mexican H7N3 highly pathogenic avian influenza viruses in poultry
Source: PLoS One. 2019 Sep 12;14(9):e0222457. doi: 10.1371/journal.pone.0222457 (PMC6742402; doi:10.1371/journal.pone.0222457)
Supplement: S2 Fig — (PDF) [file pone.0222457.s002.pdf]

Supplementary Figures 2. Time-scaled phylogenetic trees of the H7N3 HPAI and related wild bird viruses with bars to each node to display 95% HPD of the height

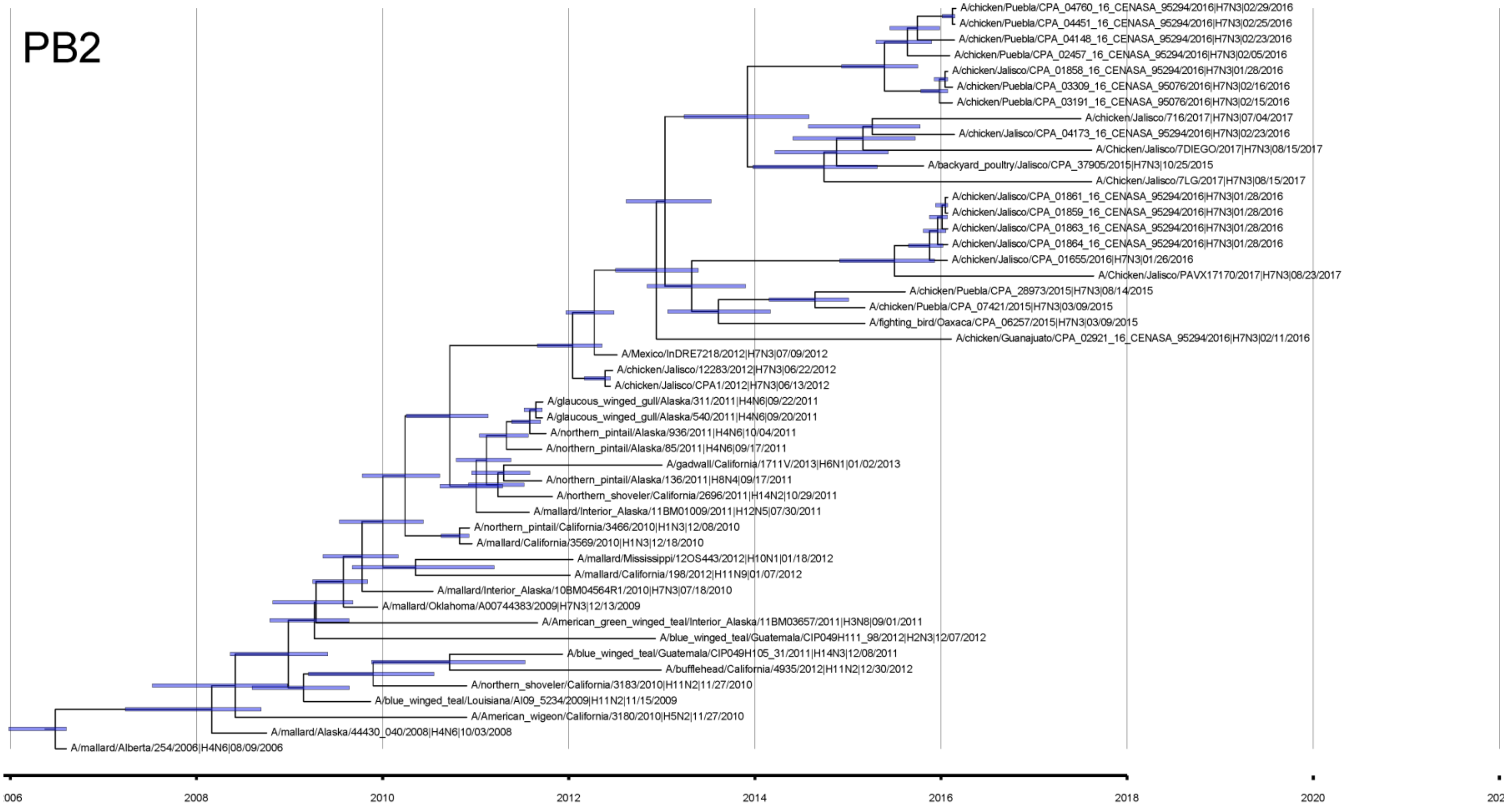

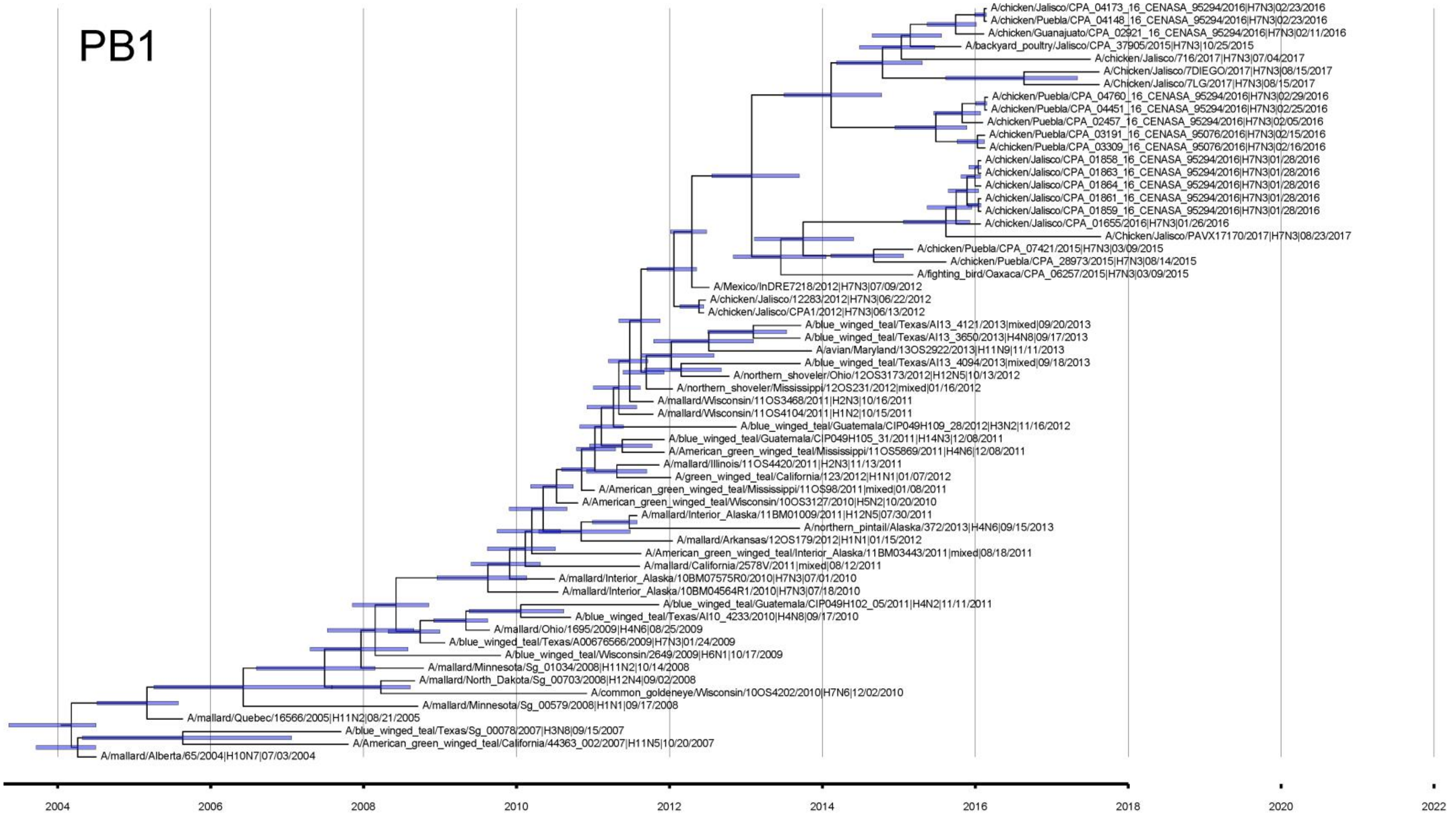
PB1
A/chicken/Jalisco/CPA_04173_16_CENASA_95294/2016|H7N3|02/23/2016
A/chicken/Puebla/CPA_04148_16_CENASA_95294/2016|H7N3|02/23/2016
A/chicken/Guanajuato/CPA_02921_16_CENASA_95294/2016|H7N3|02/11/2016
A/backyard_poultry/Jalisco/CPA_37905/2015|H7N3|10/25/2015
A/chicken/Jalisco/716/2017|H7N3|07/04/2017
A/Chicken/Jalisco/7DIEGO/2017|H7N3|08/15/2017
A/Chicken/Jalisco/7LG/2017|H7N3|08/15/2017
A/chicken/Puebla/CPA_04760_16_CENASA_95294/2016|H7N3|02/29/2016
A/chicken/Puebla/CPA_04451_16_CENASA_95294/2016|H7N3|02/25/2016
A/chicken/Puebla/CPA_02457_16_CENASA_95294/2016|H7N3|02/05/2016
A/chicken/Puebla/CPA_03191_16_CENASA_95076/2016|H7N3|02/15/2016
A/chicken/Puebla/CPA_03309_16_CENASA_95076/2016|H7N3|02/16/2016
A/chicken/Jalisco/CPA_01858_16_CENASA_95294/2016|H7N3|01/28/2016
A/chicken/Jalisco/CPA_01863_16_CENASA_95294/2016|H7N3|01/28/2016
A/chicken/Jalisco/CPA_01864_16_CENASA_95294/2016|H7N3|01/28/2016
A/chicken/Jalisco/CPA_01861_16_CENASA_95294/2016|H7N3|01/28/2016
A/chicken/Jalisco/CPA_01859_16_CENASA_95294/2016|H7N3|01/28/2016
A/chicken/Jalisco/CPA_01655/2016|H7N3|01/26/2016
A/Chicken/Jalisco/PAVX17170/2017|H7N3|08/23/2017
A/chicken/Puebla/CPA_07421/2015|H7N3|03/09/2015
A/chicken/Puebla/CPA_28973/2015|H7N3|08/14/2015
A/fighting_bird/Oaxaca/CPA_06257/2015|H7N3|03/09/2015
A/Mexico/InDRE7218/2012|H7N3|07/09/2012
A/chicken/Jalisco/12283/2012|H7N3|06/22/2012
A/chicken/Jalisco/CPA1/2012|H7N3|06/13/2012
A/blue_winged_teal/Texas/AI13_4121/2013|mixed|09/20/2013
A/blue_winged_teal/Texas/AI13_3650/2013|H4N8|09/17/2013
A/avian/Maryland/13OS2922/2013|H11N9|11/11/2013
A/blue_winged_teal/Texas/AI13_4094/2013|mixed|09/18/2013
A/northern_shoveler/Ohio/12OS3173/2012|H12N5|10/13/2012
A/northern_shoveler/Mississippi/12OS231/2012|mixed|01/16/2012
A/mallard/Wisconsin/11OS3468/2011|H2N3|10/16/2011
A/mallard/Wisconsin/11OS4104/2011|H1N2|10/15/2011
A/blue_winged_teal/Guatemala/CIP049H109_28/2012|H3N2|11/16/2012
A/blue_winged_teal/Guatemala/CIP049H105_31/2011|H14N3|12/08/2011
A/American_green_winged_teal/Mississippi/11OS5869/2011|H4N6|12/08/2011
A/mallard/Illinois/11OS4420/2011|H2N3|11/13/2011
A/green_winged_teal/California/123/2012|H1N1|01/07/2012
A/American_green_winged_teal/Mississippi/11OS98/2011|mixed|01/08/2011
A/American_green_winged_teal/Wisconsin/10OS3127/2010|H5N2|10/20/2010
A/mallard/Interior_Alaska/11BM01009/2011|H12N5|07/30/2011
A/northern_pintail/Alaska/372/2013|H4N6|09/15/2013
A/mallard/Arkansas/12OS179/2012|H1N1|01/15/2012
A/American_green_winged_teal/Interior_Alaska/11BM03443/2011|mixed|08/18/2011
A/mallard/California/2578V/2011|mixed|08/12/2011
A/mallard/Interior_Alaska/10BM07575R0/2010|H7N3|07/01/2010
A/mallard/Interior_Alaska/10BM04564R1/2010|H7N3|07/18/2010
A/blue_winged_teal/Guatemala/CIP049H102_05/2011|H4N2|11/11/2011
A/blue_winged_teal/Texas/AI10_4233/2010|H4N8|09/17/2010
A/mallard/Ohio/1695/2009|H4N6|08/25/2009
A/blue_winged_teal/Texas/A00676566/2009|H7N3|01/24/2009
A/blue_winged_teal/Wisconsin/2649/2009|H6N1|10/17/2009
A/mallard/Minnesota/Sg_01034/2008|H11N2|10/14/2008
A/mallard/North_Dakota/Sg_00703/2008|H12N4|09/02/2008
A/common_goldeneye/Wisconsin/10OS4202/2010|H7N6|12/02/2010
A/mallard/Minnesota/Sg_00579/2008|H1N1|09/17/2008
A/mallard/Quebec/16566/2005|H11N2|08/21/2005
A/blue_winged_teal/Texas/Sg_00078/2007|H3N8|09/15/2007
A/American_green_winged_teal/California/44363_002/2007|H11N5|10/20/2007
A/mallard/Alberta/65/2004|H10N7|07/03/2004
2004
2006
2008
2010
2012
2014
2016
2018
2020
2022

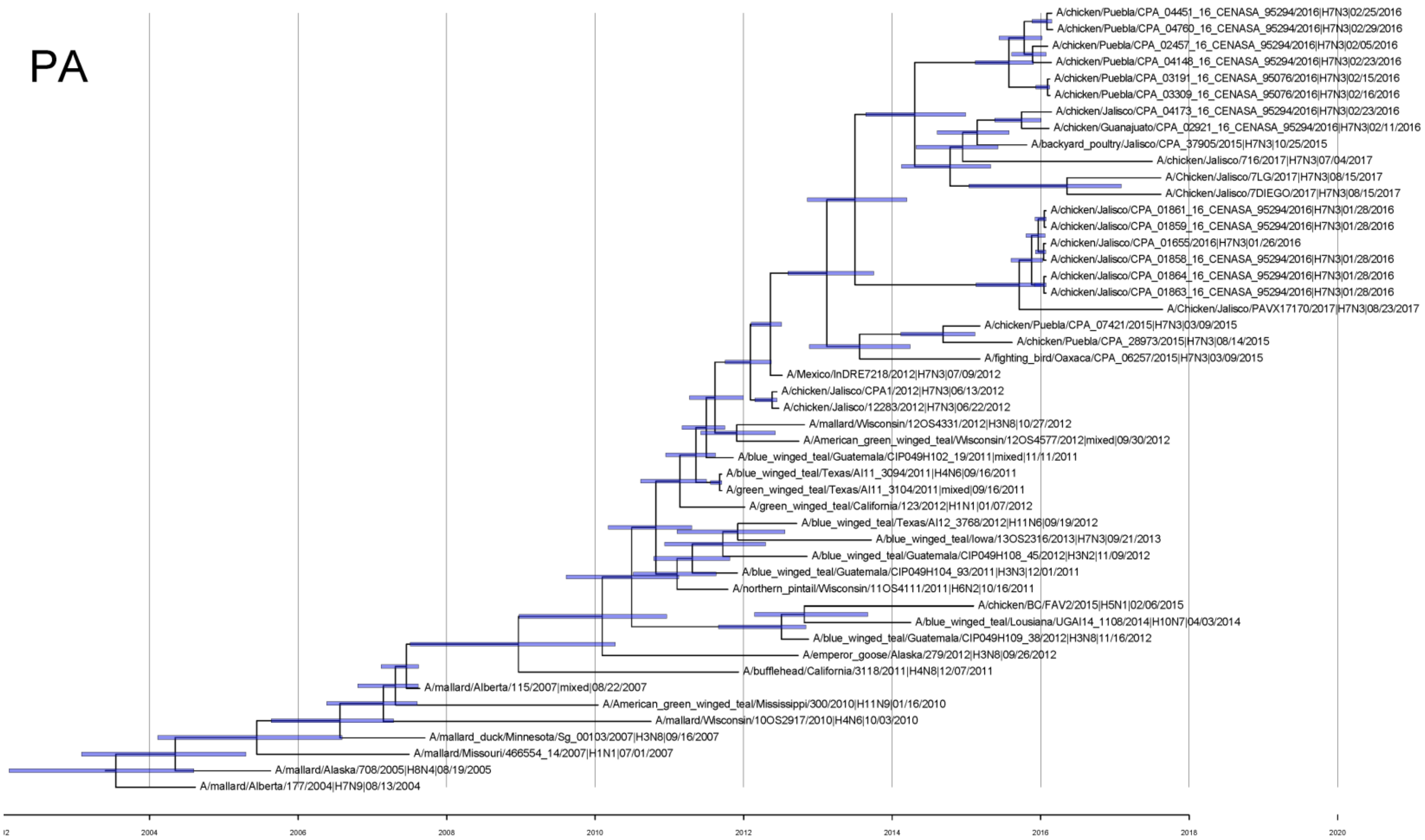

PA
A/chicken/Puebla/CPA_04451_16_CENASA_95294/2016|H7N3|02/25/2016
A/chicken/Puebla/CPA_04760_16_CENASA_95294/2016|H7N3|02/29/2016
A/chicken/Puebla/CPA_02457_16_CENASA_95294/2016|H7N3|02/05/2016
A/chicken/Puebla/CPA_04148_16_CENASA_95294/2016|H7N3|02/23/2016
A/chicken/Puebla/CPA_03191_16_CENASA_95076/2016|H7N3|02/15/2016
A/chicken/Puebla/CPA_03309_16_CENASA_95076/2016|H7N3|02/16/2016
A/chicken/Jalisco/CPA_04173_16_CENASA_95294/2016|H7N3|02/23/2016
A/chicken/Guanajuato/CPA_02921_16_CENASA_95294/2016|H7N3|02/11/2016
A/backyard_poultry/Jalisco/CPA_37905/2015|H7N3|10/25/2015
A/chicken/Jalisco/716/2017|H7N3|07/04/2017
A/Chicken/Jalisco/7LG/2017|H7N3|08/15/2017
A/Chicken/Jalisco/7DIEGO/2017|H7N3|08/15/2017
A/chicken/Jalisco/CPA_01861_16_CENASA_95294/2016|H7N3|01/28/2016
A/chicken/Jalisco/CPA_01859_16_CENASA_95294/2016|H7N3|01/28/2016
A/chicken/Jalisco/CPA_01655/2016|H7N3|01/26/2016
A/chicken/Jalisco/CPA_01858_16_CENASA_95294/2016|H7N3|01/28/2016
A/chicken/Jalisco/CPA_01864_16_CENASA_95294/2016|H7N3|01/28/2016
A/chicken/Jalisco/CPA_01863_16_CENASA_95294/2016|H7N3|01/28/2016
A/Chicken/Jalisco/PAVX17170/2017|H7N3|08/23/2017
A/chicken/Puebla/CPA_07421/2015|H7N3|03/09/2015
A/chicken/Puebla/CPA_28973/2015|H7N3|08/14/2015
A/fighting_bird/Oaxaca/CPA_06257/2015|H7N3|03/09/2015
A/Mexico/InDRE7218/2012|H7N3|07/09/2012
A/chicken/Jalisco/CPA1/2012|H7N3|06/13/2012
A/chicken/Jalisco/12283/2012|H7N3|06/22/2012
A/mallard/Wisconsin/12OS4331/2012|H3N8|10/27/2012
A/American_green_winged_teal/Wisconsin/12OS4577/2012|mixed|09/30/2012
A/blue_winged_teal/Guatemala/CIP049H102_19/2011|mixed|11/11/2011
A/blue_winged_teal/Texas/AI11_3094/2011|H4N6|09/16/2011
A/green_winged_teal/Texas/AI11_3104/2011|mixed|09/16/2011
A/green_winged_teal/California/123/2012|H1N1|01/07/2012
A/blue_winged_teal/Texas/AI12_3768/2012|H11N6|09/19/2012
A/blue_winged_teal/Iowa/13OS2316/2013|H7N3|09/21/2013
A/blue_winged_teal/Guatemala/CIP049H108_45/2012|H3N2|11/09/2012
A/blue_winged_teal/Guatemala/CIP049H104_93/2011|H3N3|12/01/2011
A/northern_pintail/Wisconsin/11OS4111/2011|H6N2|10/16/2011
A/chicken/BC/FAV2/2015|H5N1|02/06/2015
A/blue_winged_teal/Lousiana/UGAI14_1108/2014|H10N7|04/03/2014
A/blue_winged_teal/Guatemala/CIP049H109_38/2012|H3N8|11/16/2012
A/emperor_goose/Alaska/279/2012|H3N8|09/26/2012
A/bufflehead/California/3118/2011|H4N8|12/07/2011
A/mallard/Alberta/115/2007|mixed|08/22/2007
A/American_green_winged_teal/Mississippi/300/2010|H11N9|01/16/2010
A/mallard/Wisconsin/10OS2917/2010|H4N6|10/03/2010
A/mallard_duck/Minnesota/Sg_00103/2007|H3N8|09/16/2007
A/mallard/Missouri/466554_14/2007|H1N1|07/01/2007
A/mallard/Alaska/708/2005|H8N4|08/19/2005
A/mallard/Alberta/177/2004|H7N9|08/13/2004
2004
2006
2008
2010
2012
2014
2016
2018
2020
2022

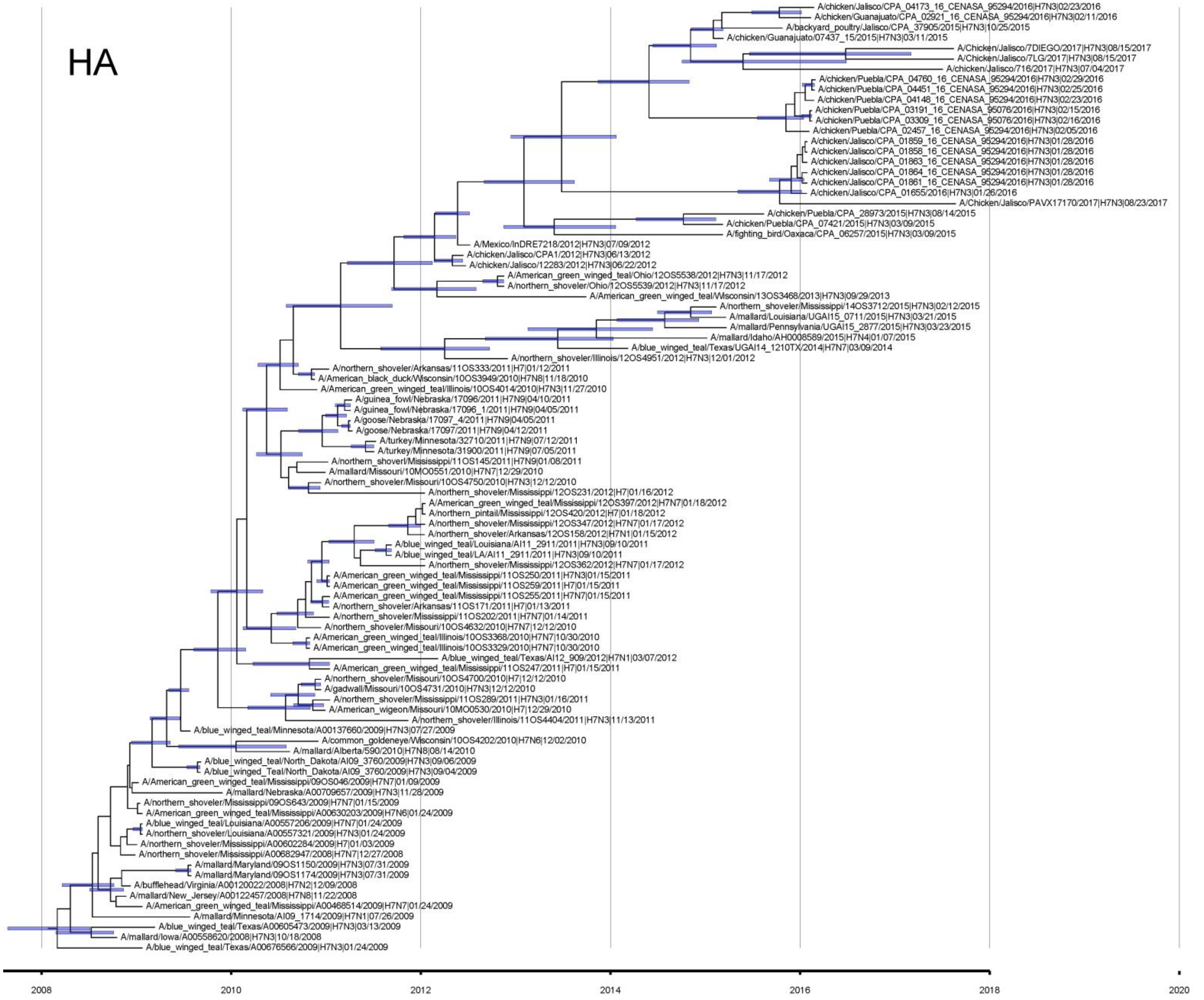
HA
A/chicken/Jalisco/CPA_04173_16_CENASA_95294/2016|H7N3|02/23/2016
A/chicken/Guanajuato/CPA_02921_16_CENASA_95294/2016|H7N3|02/11/2016
A/backyard_poultry/Jalisco/CPA_37905/2015|H7N3|10/25/2015
A/chicken/Guanajuato/07437_15/2015|H7N3|03/11/2015
A/Chicken/Jalisco/7DIEGO/2017|H7N3|08/15/2017
A/Chicken/Jalisco/7LG/2017|H7N3|08/15/2017
A/chicken/Jalisco/716/2017|H7N3|07/04/2017
A/chicken/Puebla/CPA_04760_16_CENASA_95294/2016|H7N3|02/29/2016
A/chicken/Puebla/CPA_04451_16_CENASA_95294/2016|H7N3|02/25/2016
A/chicken/Puebla/CPA_04148_16_CENASA_95294/2016|H7N3|02/23/2016
A/chicken/Puebla/CPA_03191_16_CENASA_95076/2016|H7N3|02/15/2016
A/chicken/Puebla/CPA_03309_16_CENASA_95076/2016|H7N3|02/16/2016
A/chicken/Puebla/CPA_02457_16_CENASA_95294/2016|H7N3|02/05/2016
A/chicken/Jalisco/CPA_01859_16_CENASA_95294/2016|H7N3|01/28/2016
A/chicken/Jalisco/CPA_01858_16_CENASA_95294/2016|H7N3|01/28/2016
A/chicken/Jalisco/CPA_01863_16_CENASA_95294/2016|H7N3|01/28/2016
A/chicken/Jalisco/CPA_01864_16_CENASA_95294/2016|H7N3|01/28/2016
A/chicken/Jalisco/CPA_01861_16_CENASA_95294/2016|H7N3|01/28/2016
A/chicken/Jalisco/CPA_01655/2016|H7N3|01/26/2016
A/Chicken/Jalisco/PAVX17170/2017|H7N3|08/23/2017
A/chicken/Puebla/CPA_28973/2015|H7N3|08/14/2015
A/chicken/Puebla/CPA_07421/2015|H7N3|03/09/2015
A/fighting_bird/Oaxaca/CPA_06257/2015|H7N3|03/09/2015
A/Mexico/InDRE7218/2012|H7N3|07/09/2012
A/chicken/Jalisco/CPA1/2012|H7N3|06/13/2012
A/chicken/Jalisco/12283/2012|H7N3|06/22/2012
A/American_green_winged_teal/Ohio/12OS5538/2012|H7N3|11/17/2012
A/northern_shoveler/Ohio/12OS5539/2012|H7N3|11/17/2012
A/American_green_winged_teal/Wisconsin/13OS3468/2013|H7N3|09/29/2013
A/northern_shoveler/Mississippi/14OS3712/2015|H7N3|02/12/2015
A/mallard/Louisiana/UGAI15_0711/2015|H7N3|03/21/2015
A/mallard/Pennsylvania/UGAI15_2877/2015|H7N3|03/23/2015
A/mallard/Idaho/AH0008589/2015|H7N4|01/07/2015
A/blue_winged_teal/Texas/UGAI14_1210TX/2014|H7N7|03/09/2014
A/northern_shoveler/Illinois/12OS4951/2012|H7N3|12/01/2012
A/northern_shoveler/Arkansas/11OS333/2011|H7|01/12/2011
A/American_black_duck/Wisconsin/10OS3949/2010|H7N8|11/18/2010
A/American_green_winged_teal/Illinois/10OS4014/2010|H7N3|11/27/2010
A/guinea_fowl/Nebraska/17096/2011|H7N9|04/10/2011
A/guinea_fowl/Nebraska/17096_1/2011|H7N9|04/05/2011
A/goose/Nebraska/17097_4/2011|H7N9|04/05/2011
A/goose/Nebraska/17097/2011|H7N9|04/12/2011
A/turkey/Minnesota/32710/2011|H7N9|07/12/2011
A/turkey/Minnesota/31900/2011|H7N9|07/05/2011
A/northern_shoverl/Mississippi/11OS145/2011|H7N9|01/08/2011
A/mallard/Missouri/10MO0551/2010|H7N7|12/29/2010
A/northern_shoveler/Missouri/10OS4750/2010|H7N3|12/12/2010
A/northern_shoveler/Mississippi/12OS231/2012|H7|01/16/2012
A/American_green_winged_teal/Mississippi/12OS397/2012|H7N7|01/18/2012
A/northern_pintail/Mississippi/12OS420/2012|H7|01/18/2012
A/northern_shoveler/Mississippi/12OS347/2012|H7N7|01/17/2012
A/northern_shoveler/Arkansas/12OS158/2012|H7N1|01/15/2012
A/blue_winged_teal/Louisiana/AI11_2911/2011|H7N3|09/10/2011
A/blue_winged_teal/LA/AI11_2911/2011|H7N3|09/10/2011
A/northern_shoveler/Mississippi/12OS362/2012|H7N7|01/17/2012
A/American_green_winged_teal/Mississippi/11OS250/2011|H7N3|01/15/2011
A/American_green_winged_teal/Mississippi/11OS259/2011|H7|01/15/2011
A/American_green_winged_teal/Mississippi/11OS255/2011|H7N7|01/15/2011
A/northern_shoveler/Arkansas/11OS171/2011|H7|01/13/2011
A/northern_shoveler/Mississippi/11OS202/2011|H7N7|01/14/2011
A/northern_shoveler/Missouri/10OS4632/2010|H7N7|12/12/2010
A/American_green_winged_teal/Illinois/10OS3368/2010|H7N7|10/30/2010
A/American_green_winged_teal/Illinois/10OS3329/2010|H7N7|10/30/2010
A/blue_winged_teal/Texas/AI12_909/2012|H7N1|03/07/2012
A/American_green_winged_teal/Mississippi/11OS247/2011|H7|01/15/2011
A/northern_shoveler/Missouri/10OS4700/2010|H7|12/12/2010
A/gadwall/Missouri/10OS4731/2010|H7N3|12/12/2010
A/northern_shoveler/Mississippi/11OS289/2011|H7N3|01/16/2011
A/American_wigeon/Missouri/10MO0530/2010|H7|12/29/2010
A/northern_shoveler/Illinois/11OS4404/2011|H7N3|11/13/2011
A/blue_winged_teal/Minnesota/A00137660/2009|H7N3|07/27/2009
A/common_goldeneye/Wisconsin/10OS4202/2010|H7N6|12/02/2010
A/mallard/Alberta/590/2010|H7N8|08/14/2010
A/blue_winged_teal/North_Dakota/AI09_3760/2009|H7N3|09/06/2009
A/blue_winged_Teal/North_Dakota/AI09_3760/2009|H7N3|09/04/2009
A/American_green_winged_teal/Mississippi/09OS046/2009|H7N7|01/09/2009
A/mallard/Nebraska/A00709657/2009|H7N3|11/28/2009
A/northern_shoveler/Mississippi/09OS643/2009|H7N7|01/15/2009
A/American_green_winged_teal/Mississippi/A00630203/2009|H7N6|01/24/2009
A/blue_winged_teal/Louisiana/A00557206/2009|H7N7|01/24/2009
A/northern_shoveler/Louisiana/A00557321/2009|H7N3|01/24/2009
A/northern_shoveler/Mississippi/A00602284/2009|H7|01/03/2009
A/northern_shoveler/Mississippi/A00682947/2008|H7N7|12/27/2008
A/mallard/Maryland/09OS1150/2009|H7N3|07/31/2009
A/mallard/Maryland/09OS1174/2009|H7N3|07/31/2009
A/bufflehead/Virginia/A00120022/2008|H7N2|12/09/2008
A/mallard/New_Jersey/A00122457/2008|H7N8|11/22/2008
A/American_green_winged_teal/Mississippi/A00468514/2009|H7N7|01/24/2009
A/mallard/Minnesota/AI09_1714/2009|H7N1|07/26/2009
A/blue_winged_teal/Texas/A00605473/2009|H7N3|03/13/2009
A/mallard/Iowa/A00558620/2008|H7N3|10/18/2008
A/blue_winged_teal/Texas/A00676566/2009|H7N3|01/24/2009
2008
2010
2012
2014
2016
2018
2020

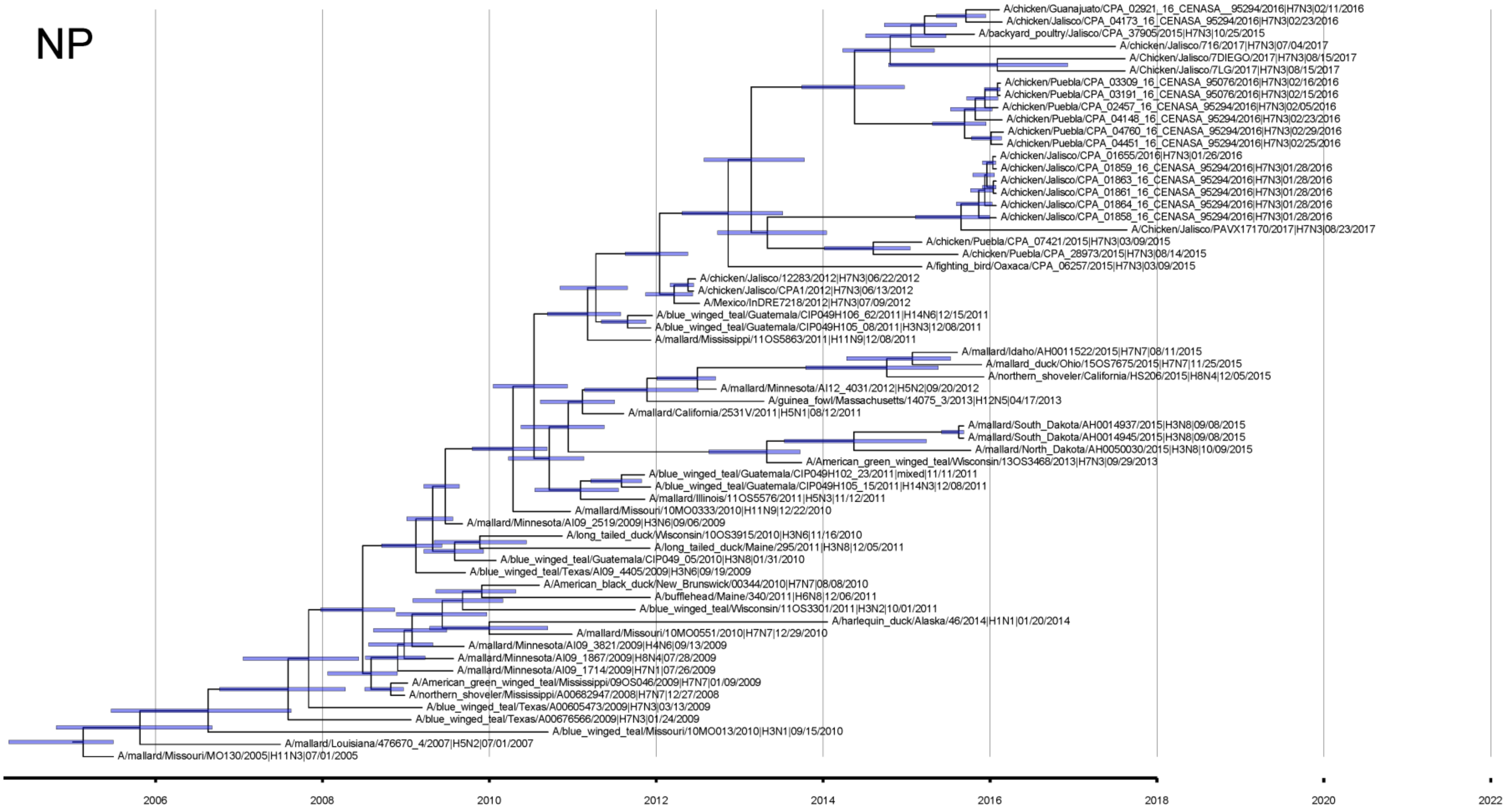

NP
A/chicken/Guanajuato/CPA_02921_16_CENASA__95294/2016|H7N3|02/11/2016
A/chicken/Jalisco/CPA_04173_16_CENASA_95294/2016|H7N3|02/23/2016
A/backyard_poultry/Jalisco/CPA_37905/2015|H7N3|10/25/2015
A/chicken/Jalisco/716/2017|H7N3|07/04/2017
A/Chicken/Jalisco/7DIEGO/2017|H7N3|08/15/2017
A/Chicken/Jalisco/7LG/2017|H7N3|08/15/2017
A/chicken/Puebla/CPA_03309_16_CENASA_95076/2016|H7N3|02/16/2016
A/chicken/Puebla/CPA_03191_16_CENASA_95076/2016|H7N3|02/15/2016
A/chicken/Puebla/CPA_02457_16_CENASA_95294/2016|H7N3|02/05/2016
A/chicken/Puebla/CPA_04148_16_CENASA_95294/2016|H7N3|02/23/2016
A/chicken/Puebla/CPA_04760_16_CENASA_95294/2016|H7N3|02/29/2016
A/chicken/Puebla/CPA_04451_16_CENASA_95294/2016|H7N3|02/25/2016
A/chicken/Jalisco/CPA_01655/2016|H7N3|01/26/2016
A/chicken/Jalisco/CPA_01859_16_CENASA_95294/2016|H7N3|01/28/2016
A/chicken/Jalisco/CPA_01863_16_CENASA_95294/2016|H7N3|01/28/2016
A/chicken/Jalisco/CPA_01861_16_CENASA_95294/2016|H7N3|01/28/2016
A/chicken/Jalisco/CPA_01864_16_CENASA_95294/2016|H7N3|01/28/2016
A/chicken/Jalisco/CPA_01858_16_CENASA_95294/2016|H7N3|01/28/2016
A/Chicken/Jalisco/PAVX17170/2017|H7N3|08/23/2017
A/chicken/Puebla/CPA_07421/2015|H7N3|03/09/2015
A/chicken/Puebla/CPA_28973/2015|H7N3|08/14/2015
A/fighting_bird/Oaxaca/CPA_06257/2015|H7N3|03/09/2015
A/chicken/Jalisco/12283/2012|H7N3|06/22/2012
A/chicken/Jalisco/CPA1/2012|H7N3|06/13/2012
A/Mexico/InDRE7218/2012|H7N3|07/09/2012
A/blue_winged_teal/Guatemala/CIP049H106_62/2011|H14N6|12/15/2011
A/blue_winged_teal/Guatemala/CIP049H105_08/2011|H3N3|12/08/2011
A/mallard/Mississippi/11OS5863/2011|H11N9|12/08/2011
A/mallard/Idaho/AH0011522/2015|H7N7|08/11/2015
A/mallard_duck/Ohio/15OS7675/2015|H7N7|11/25/2015
A/northern_shoveler/California/HS206/2015|H8N4|12/05/2015
A/mallard/Minnesota/AI12_4031/2012|H5N2|09/20/2012
A/guinea_fowl/Massachusetts/14075_3/2013|H12N5|04/17/2013
A/mallard/California/2531V/2011|H5N1|08/12/2011
A/mallard/South_Dakota/AH0014937/2015|H3N8|09/08/2015
A/mallard/South_Dakota/AH0014945/2015|H3N8|09/08/2015
A/mallard/North_Dakota/AH0050030/2015|H3N8|10/09/2015
A/American_green_winged_teal/Wisconsin/13OS3468/2013|H7N3|09/29/2013
A/blue_winged_teal/Guatemala/CIP049H102_23/2011|mixed|11/11/2011
A/blue_winged_teal/Guatemala/CIP049H105_15/2011|H14N3|12/08/2011
A/mallard/Illinois/11OS5576/2011|H5N3|11/12/2011
A/mallard/Missouri/10MO0333/2010|H11N9|12/22/2010
A/mallard/Minnesota/AI09_2519/2009|H3N6|09/06/2009
A/long_tailed_duck/Wisconsin/10OS3915/2010|H3N6|11/16/2010
A/long_tailed_duck/Maine/295/2011|H3N8|12/05/2011
A/blue_winged_teal/Guatemala/CIP049_05/2010|H3N8|01/31/2010
A/blue_winged_teal/Texas/AI09_4405/2009|H3N6|09/19/2009
A/American_black_duck/New_Brunswick/00344/2010|H7N7|08/08/2010
A/bufflehead/Maine/340/2011|H6N8|12/06/2011
A/blue_winged_teal/Wisconsin/11OS3301/2011|H3N2|10/01/2011
A/harlequin_duck/Alaska/46/2014|H1N1|01/20/2014
A/mallard/Missouri/10MO0551/2010|H7N7|12/29/2010
A/mallard/Minnesota/AI09_3821/2009|H4N6|09/13/2009
A/mallard/Minnesota/AI09_1867/2009|H8N4|07/28/2009
A/mallard/Minnesota/AI09_1714/2009|H7N1|07/26/2009
A/American_green_winged_teal/Mississippi/09OS046/2009|H7N7|01/09/2009
A/northern_shoveler/Mississippi/A00682947/2008|H7N7|12/27/2008
A/blue_winged_teal/Texas/A00605473/2009|H7N3|03/13/2009
A/blue_winged_teal/Texas/A00676566/2009|H7N3|01/24/2009
A/blue_winged_teal/Missouri/10MO013/2010|H3N1|09/15/2010
A/mallard/Louisiana/476670_4/2007|H5N2|07/01/2007
A/mallard/Missouri/MO130/2005|H11N3|07/01/2005
2006
2008
2010
2012
2014
2016
2018
2020
2022

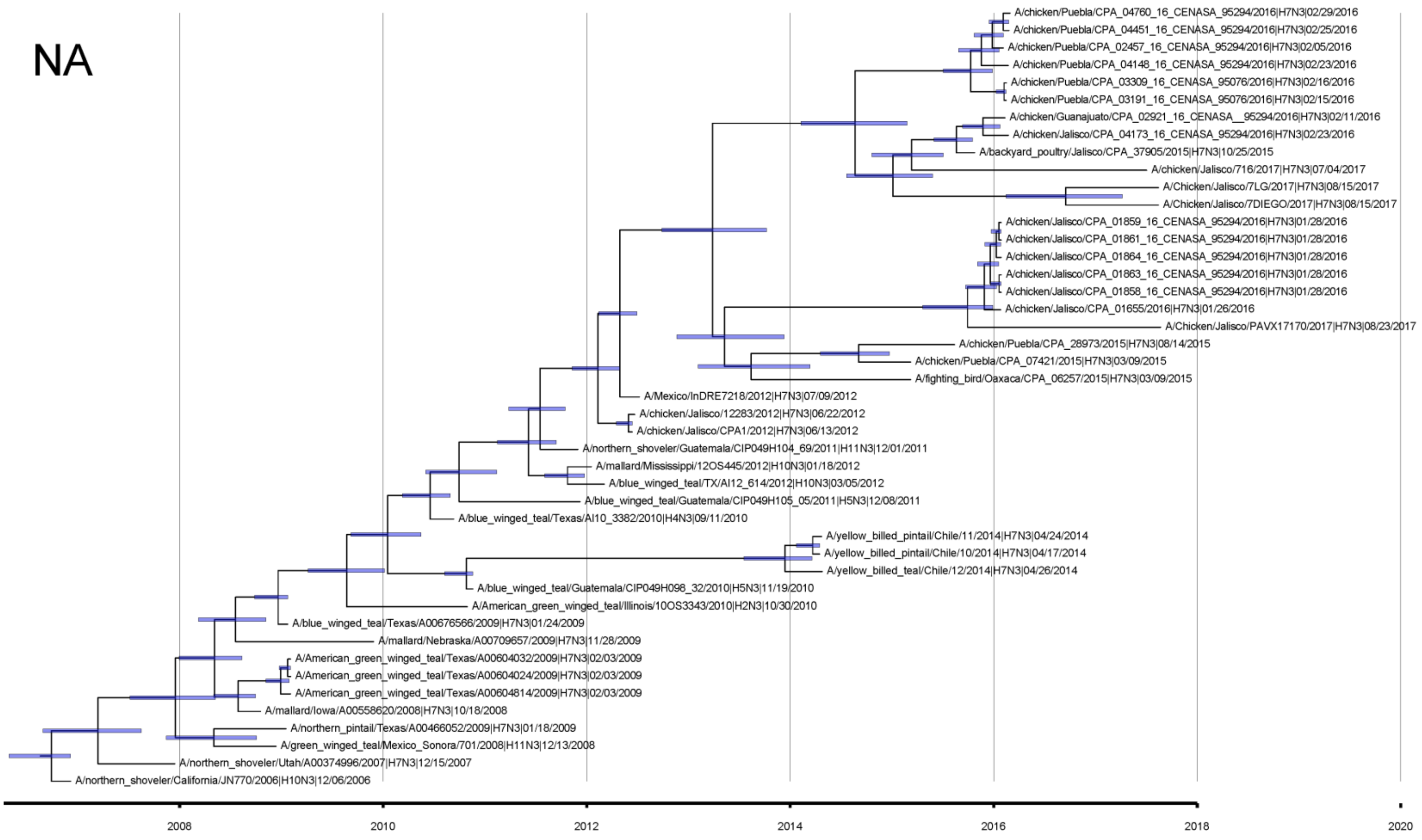
NA
A/chicken/Puebla/CPA_04760_16_CENASA_95294/2016|H7N3|02/29/2016
A/chicken/Puebla/CPA_04451_16_CENASA_95294/2016|H7N3|02/25/2016
A/chicken/Puebla/CPA_02457_16_CENASA_95294/2016|H7N3|02/05/2016
A/chicken/Puebla/CPA_04148_16_CENASA_95294/2016|H7N3|02/23/2016
A/chicken/Puebla/CPA_03309_16_CENASA_95076/2016|H7N3|02/16/2016
A/chicken/Puebla/CPA_03191_16_CENASA_95076/2016|H7N3|02/15/2016
A/chicken/Guanajuato/CPA_02921_16_CENASA__95294/2016|H7N3|02/11/2016
A/chicken/Jalisco/CPA_04173_16_CENASA_95294/2016|H7N3|02/23/2016
A/backyard_poultry/Jalisco/CPA_37905/2015|H7N3|10/25/2015
A/chicken/Jalisco/716/2017|H7N3|07/04/2017
A/Chicken/Jalisco/7LG/2017|H7N3|08/15/2017
A/Chicken/Jalisco/7DIEGO/2017|H7N3|08/15/2017
A/chicken/Jalisco/CPA_01859_16_CENASA_95294/2016|H7N3|01/28/2016
A/chicken/Jalisco/CPA_01861_16_CENASA_95294/2016|H7N3|01/28/2016
A/chicken/Jalisco/CPA_01864_16_CENASA_95294/2016|H7N3|01/28/2016
A/chicken/Jalisco/CPA_01863_16_CENASA_95294/2016|H7N3|01/28/2016
A/chicken/Jalisco/CPA_01858_16_CENASA_95294/2016|H7N3|01/28/2016
A/chicken/Jalisco/CPA_01655/2016|H7N3|01/26/2016
A/Chicken/Jalisco/PAVX17170/2017|H7N3|08/23/2017
A/chicken/Puebla/CPA_28973/2015|H7N3|08/14/2015
A/chicken/Puebla/CPA_07421/2015|H7N3|03/09/2015
A/fighting_bird/Oaxaca/CPA_06257/2015|H7N3|03/09/2015
A/Mexico/InDRE7218/2012|H7N3|07/09/2012
A/chicken/Jalisco/12283/2012|H7N3|06/22/2012
A/chicken/Jalisco/CPA1/2012|H7N3|06/13/2012
A/northern_shoveler/Guatemala/CIP049H104_69/2011|H11N3|12/01/2011
A/mallard/Mississippi/12OS445/2012|H10N3|01/18/2012
A/blue_winged_teal/TX/AI12_614/2012|H10N3|03/05/2012
A/blue_winged_teal/Guatemala/CIP049H105_05/2011|H5N3|12/08/2011
A/blue_winged_teal/Texas/AI10_3382/2010|H4N3|09/11/2010
A/yellow_billed_pintail/Chile/11/2014|H7N3|04/24/2014
A/yellow_billed_pintail/Chile/10/2014|H7N3|04/17/2014
A/yellow_billed_teal/Chile/12/2014|H7N3|04/26/2014
A/blue_winged_teal/Guatemala/CIP049H098_32/2010|H5N3|11/19/2010
A/American_green_winged_teal/Illinois/10OS3343/2010|H2N3|10/30/2010
A/blue_winged_teal/Texas/A00676566/2009|H7N3|01/24/2009
A/mallard/Nebraska/A00709657/2009|H7N3|11/28/2009
A/American_green_winged_teal/Texas/A00604032/2009|H7N3|02/03/2009
A/American_green_winged_teal/Texas/A00604024/2009|H7N3|02/03/2009
A/American_green_winged_teal/Texas/A00604814/2009|H7N3|02/03/2009
A/mallard/Iowa/A00558620/2008|H7N3|10/18/2008
A/northern_pintail/Texas/A00466052/2009|H7N3|01/18/2009
A/green_winged_teal/Mexico_Sonora/701/2008|H11N3|12/13/2008
A/northern_shoveler/Utah/A00374996/2007|H7N3|12/15/2007
A/northern_shoveler/California/JN770/2006|H10N3|12/06/2006
2008
2010
2012
2014
2016
2018
2020

M

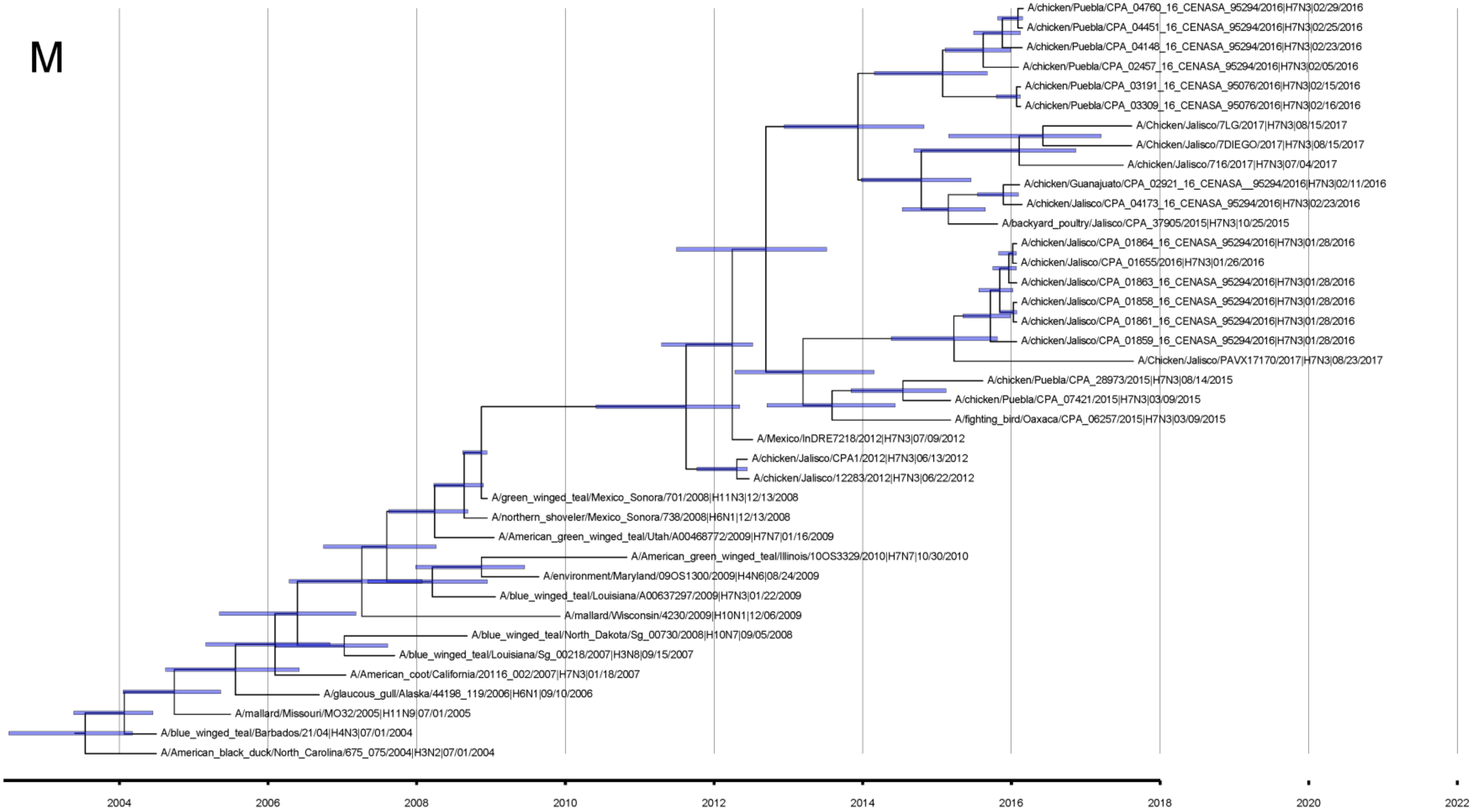

A/chicken/Puebla/CPA_04760_16_CENASA_95294/2016|H7N3|02/29/2016
A/chicken/Puebla/CPA_04451_16_CENASA_95294/2016|H7N3|02/25/2016
A/chicken/Puebla/CPA_04148_16_CENASA_95294/2016|H7N3|02/23/2016
A/chicken/Puebla/CPA_02457_16_CENASA_95294/2016|H7N3|02/05/2016
A/chicken/Puebla/CPA_03191_16_CENASA_95076/2016|H7N3|02/15/2016
A/chicken/Puebla/CPA_03309_16_CENASA_95076/2016|H7N3|02/16/2016
A/Chicken/Jalisco/7LG/2017|H7N3|08/15/2017
A/Chicken/Jalisco/7DIEGO/2017|H7N3|08/15/2017
A/chicken/Jalisco/716/2017|H7N3|07/04/2017
A/chicken/Guanajuato/CPA_02921_16_CENASA__95294/2016|H7N3|02/11/2016
A/chicken/Jalisco/CPA_04173_16_CENASA_95294/2016|H7N3|02/23/2016
A/backyard_poultry/Jalisco/CPA_37905/2015|H7N3|10/25/2015
A/chicken/Jalisco/CPA_01864_16_CENASA_95294/2016|H7N3|01/28/2016
A/chicken/Jalisco/CPA_01655/2016|H7N3|01/26/2016
A/chicken/Jalisco/CPA_01863_16_CENASA_95294/2016|H7N3|01/28/2016
A/chicken/Jalisco/CPA_01858_16_CENASA_95294/2016|H7N3|01/28/2016
A/chicken/Jalisco/CPA_01861_16_CENASA_95294/2016|H7N3|01/28/2016
A/chicken/Jalisco/CPA_01859_16_CENASA_95294/2016|H7N3|01/28/2016
A/Chicken/Jalisco/PAVX17170/2017|H7N3|08/23/2017
A/chicken/Puebla/CPA_28973/2015|H7N3|08/14/2015
A/chicken/Puebla/CPA_07421/2015|H7N3|03/09/2015
A/fighting_bird/Oaxaca/CPA_06257/2015|H7N3|03/09/2015
A/Mexico/InDRE7218/2012|H7N3|07/09/2012
A/chicken/Jalisco/CPA1/2012|H7N3|06/13/2012
A/chicken/Jalisco/12283/2012|H7N3|06/22/2012
A/green_winged_teal/Mexico_Sonora/701/2008|H11N3|12/13/2008
A/northern_shoveler/Mexico_Sonora/738/2008|H6N1|12/13/2008
A/American_green_winged_teal/Utah/A00468772/2009|H7N7|01/16/2009
A/American_green_winged_teal/Illinois/10OS3329/2010|H7N7|10/30/2010
A/environment/Maryland/09OS1300/2009|H4N6|08/24/2009
A/blue_winged_teal/Louisiana/A00637297/2009|H7N3|01/22/2009
A/mallard/Wisconsin/4230/2009|H10N1|12/06/2009
A/blue_winged_teal/North_Dakota/Sg_00730/2008|H10N7|09/05/2008
A/blue_winged_teal/Louisiana/Sg_00218/2007|H3N8|09/15/2007
A/American_coot/California/20116_002/2007|H7N3|01/18/2007
A/glaucous_gull/Alaska/44198_119/2006|H6N1|09/10/2006
A/mallard/Missouri/MO32/2005|H11N9|07/01/2005
A/blue_winged_teal/Barbados/21/04|H4N3|07/01/2004
A/American_black_duck/North_Carolina/675_075/2004|H3N2|07/01/2004
2004
2006
2008
2010
2012
2014
2016
2018
2020
2022

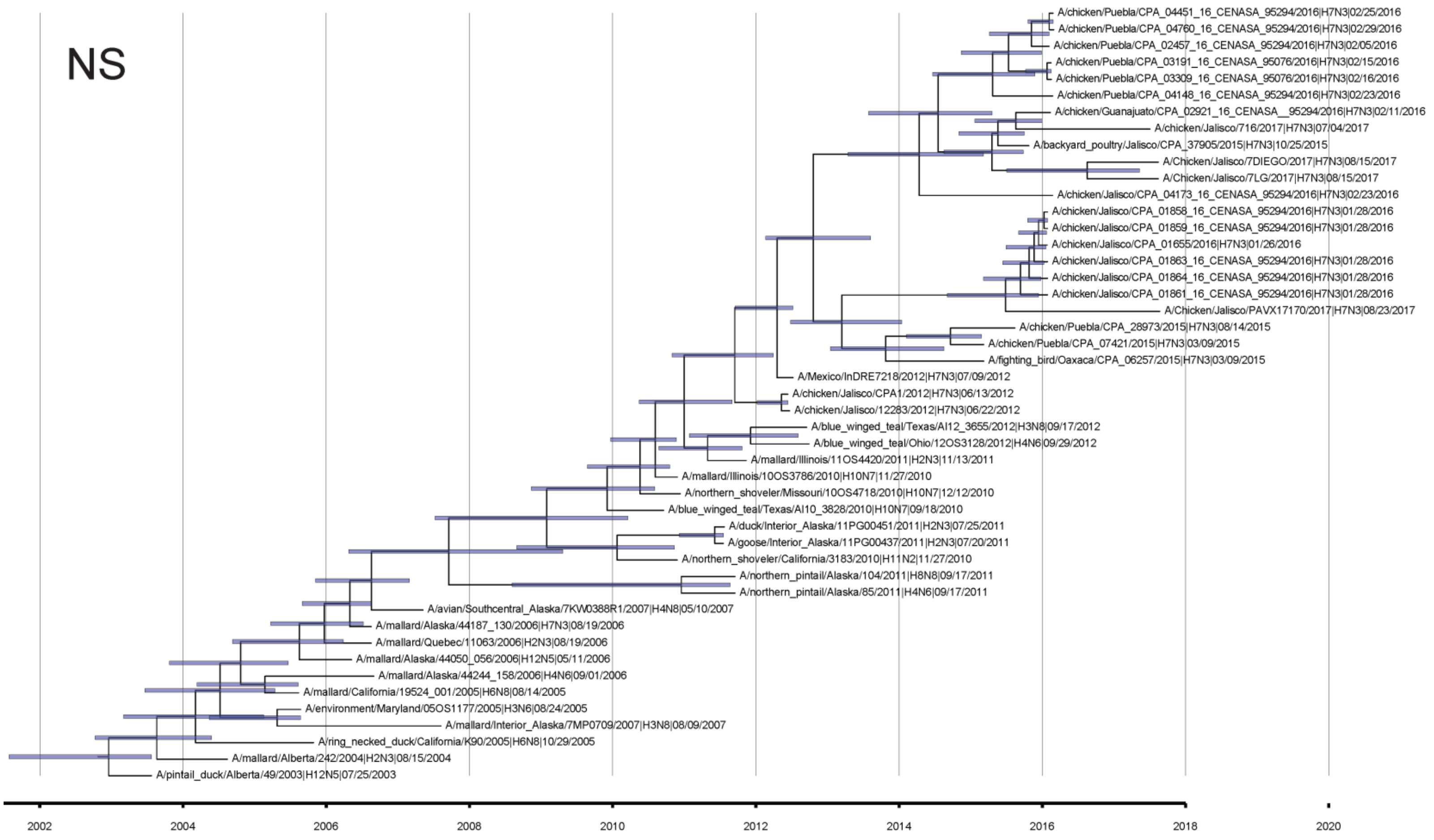
NS
A/chicken/Puebla/CPA_04451_16_CENASA_95294/2016|H7N3|02/25/2016
A/chicken/Puebla/CPA_04760_16_CENASA_95294/2016|H7N3|02/29/2016
A/chicken/Puebla/CPA_02457_16_CENASA_95294/2016|H7N3|02/05/2016
A/chicken/Puebla/CPA_03191_16_CENASA_95076/2016|H7N3|02/15/2016
A/chicken/Puebla/CPA_03309_16_CENASA_95076/2016|H7N3|02/16/2016
A/chicken/Puebla/CPA_04148_16_CENASA_95294/2016|H7N3|02/23/2016
A/chicken/Guanajuato/CPA_02921_16_CENASA__95294/2016|H7N3|02/11/2016
A/chicken/Jalisco/716/2017|H7N3|07/04/2017
A/backyard_poultry/Jalisco/CPA_37905/2015|H7N3|10/25/2015
A/Chicken/Jalisco/7DIEGO/2017|H7N3|08/15/2017
A/Chicken/Jalisco/7LG/2017|H7N3|08/15/2017
A/chicken/Jalisco/CPA_04173_16_CENASA_95294/2016|H7N3|02/23/2016
A/chicken/Jalisco/CPA_01858_16_CENASA_95294/2016|H7N3|01/28/2016
A/chicken/Jalisco/CPA_01859_16_CENASA_95294/2016|H7N3|01/28/2016
A/chicken/Jalisco/CPA_01655/2016|H7N3|01/26/2016
A/chicken/Jalisco/CPA_01863_16_CENASA_95294/2016|H7N3|01/28/2016
A/chicken/Jalisco/CPA_01864_16_CENASA_95294/2016|H7N3|01/28/2016
A/chicken/Jalisco/CPA_01861_16_CENASA_95294/2016|H7N3|01/28/2016
A/Chicken/Jalisco/PAVX17170/2017|H7N3|08/23/2017
A/chicken/Puebla/CPA_28973/2015|H7N3|08/14/2015
A/chicken/Puebla/CPA_07421/2015|H7N3|03/09/2015
A/fighting_bird/Oaxaca/CPA_06257/2015|H7N3|03/09/2015
A/Mexico/InDRE7218/2012|H7N3|07/09/2012
A/chicken/Jalisco/CPA1/2012|H7N3|06/13/2012
A/chicken/Jalisco/12283/2012|H7N3|06/22/2012
A/blue_winged_teal/Texas/AI12_3655/2012|H3N8|09/17/2012
A/blue_winged_teal/Ohio/12OS3128/2012|H4N6|09/29/2012
A/mallard/Illinois/11OS4420/2011|H2N3|11/13/2011
A/mallard/Illinois/10OS3786/2010|H10N7|11/27/2010
A/northern_shoveler/Missouri/10OS4718/2010|H10N7|12/12/2010
A/blue_winged_teal/Texas/AI10_3828/2010|H10N7|09/18/2010
A/duck/Interior_Alaska/11PG00451/2011|H2N3|07/25/2011
A/goose/Interior_Alaska/11PG00437/2011|H2N3|07/20/2011
A/northern_shoveler/California/3183/2010|H11N2|11/27/2010
A/northern_pintail/Alaska/104/2011|H8N8|09/17/2011
A/northern_pintail/Alaska/85/2011|H4N6|09/17/2011
A/avian/Southcentral_Alaska/7KW0388R1/2007|H4N8|05/10/2007
A/mallard/Alaska/44187_130/2006|H7N3|08/19/2006
A/mallard/Quebec/11063/2006|H2N3|08/19/2006
A/mallard/Alaska/44050_056/2006|H12N5|05/11/2006
A/mallard/Alaska/44244_158/2006|H4N6|09/01/2006
A/mallard/California/19524_001/2005|H6N8|08/14/2005
A/environment/Maryland/05OS1177/2005|H3N6|08/24/2005
A/mallard/Interior_Alaska/7MP0709/2007|H3N8|08/09/2007
A/ring_necked_duck/California/K90/2005|H6N8|10/29/2005
A/mallard/Alberta/242/2004|H2N3|08/15/2004
A/pintail_duck/Alberta/49/2003|H12N5|07/25/2003
2002
2004
2006
2008
2010
2012
2014
2016
2018
2020
2022
