# Supplementary figures and images for: Rapid evolution of Mexican H7N3 highly pathogenic avian influenza viruses in poultry
Source: PLoS One. 2019 Sep 12;14(9):e0222457. doi: 10.1371/journal.pone.0222457 (PMC6742402; doi:10.1371/journal.pone.0222457)

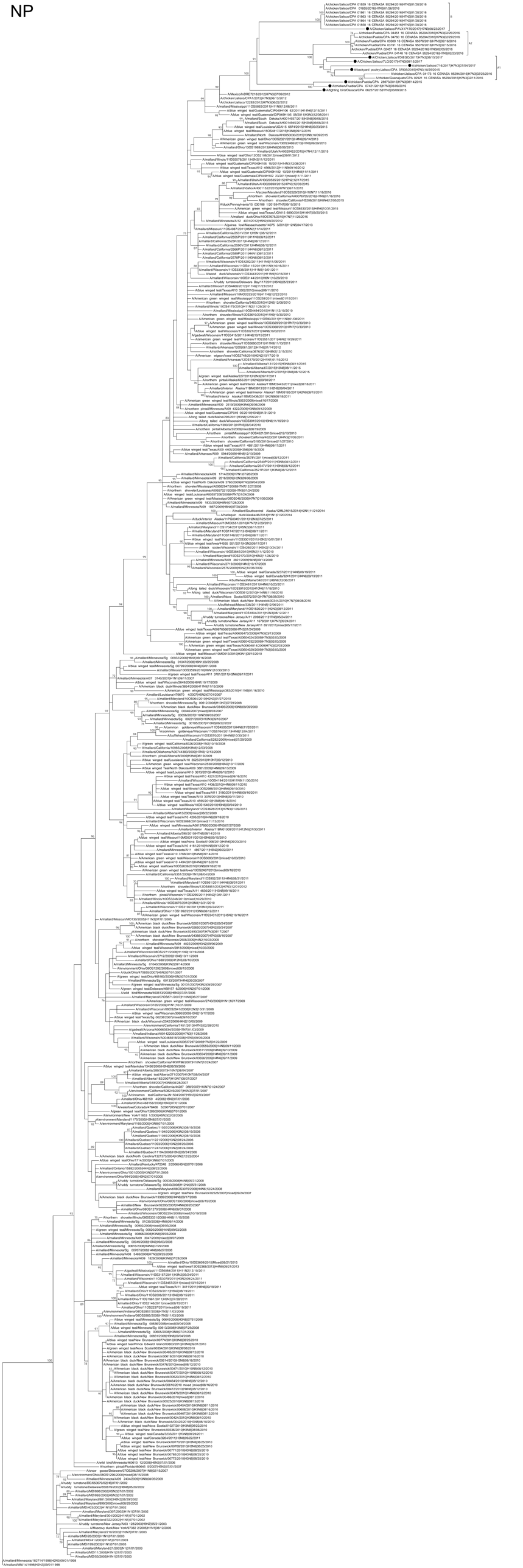

A/mallard/Missouri/182714/1998/H2N3/09/01/1998  
A/mallard/MN/14/1998/H2N3/09/01/1998

0.002



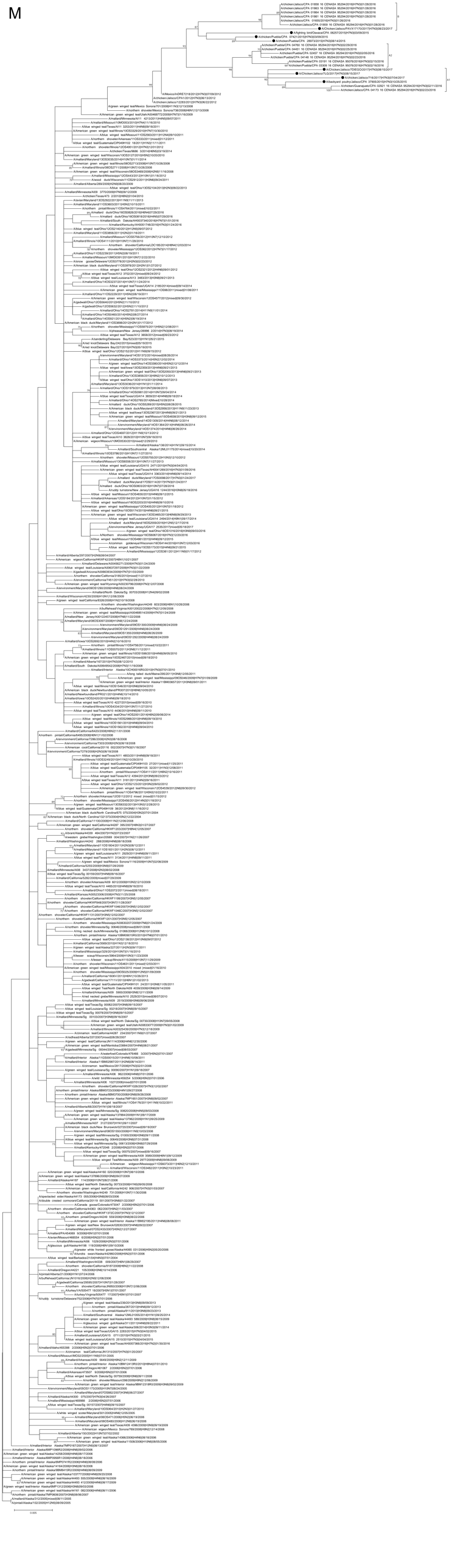

Supplement: S4 Fig — Closed circles are viruses isolated and sequenced in this study. Brackets indicate genetic cluster of the H7N3 HPAI. (PDF) [file pone.0222457.s004.pdf]
