# Supplementary material for: Rapid evolution of Mexican H7N3 highly pathogenic avian influenza viruses in poultry
Source: PLoS One. 2019 Sep 12;14(9):e0222457. doi: 10.1371/journal.pone.0222457 (PMC6742402; doi:10.1371/journal.pone.0222457)
Supplement: S1 Table — (DOCX) [file pone.0222457.s005.docx]

Supplementary Table 1. The H7N3 HPAI viruses isolated in this study (strain, collection date and Genbank accession number)

| Strain | Date | Type of farm | Accession number |
| --- | --- | --- | --- |
| A/fighting bird/Oaxaca/CPA 06257/2015 | 03/09/2015 | Backyard | MK559330-MK559337 |
| A/Chicken/Puebla/CPA 07421/2015 | 03/09/2015 | Backyard | MK559785-MK559792 |
| A/Chicken/Puebla/CPA 28973/2015 | 08/14/2015 | Backyard | MK027376- MK027383 |
| A/Backyard poultry/Jalisco/CPA 37905/2015 | 10/25/2015 | Backyard | MK559349-MK559356 |
| A/Chicken/Jalisco/716/2017 | 07/04/2017 | Backyard | MK559297-MK559304 |
| A/Chicken/Jalisco/7LG/2017 | 08/15/2017 | Backyard | MK559305-MK559312 |
| A/Chicken/Jalisco/7DIEGO/2017 | 08/18/2017 | Backyard | MK558923-MK558930 |
| A/Chicken/Jalisco/PAVX17170/2017 | 08/23/2017 | Backyard | MK559385-MK559392 |
