# Supplementary material for: Rapid evolution of Mexican H7N3 highly pathogenic avian influenza viruses in poultry
Source: PLoS One. 2019 Sep 12;14(9):e0222457. doi: 10.1371/journal.pone.0222457 (PMC6742402; doi:10.1371/journal.pone.0222457)
Supplement: S2 Table — (DOCX) [file pone.0222457.s006.docx]

Supplementary Table 2. Comparison of nucleotide similarity in percentage between the initial isolate (CPA1) and subclusters

| Segments | CPA1 - A1 | CPA1 - A2 | CPA1 - B |
| --- | --- | --- | --- |
| PB2 | 97.36 – 98.24 | 97.32 – 97.50 | 96.71 – 97.72 |
| PB1 | 96.21 – 96.96 | 96.70 – 96.92 | 95.99 – 96.87 |
| PA | 96.88 – 97.53 | 97.11 – 97.21 | 97.11 – 97.95 |
| HA | 94.94 – 96.55 | 95.95 – 96.07 | 95.18 – 96.55 |
| NP | 96.72 – 97.79 | 97.59 – 97.79 | 97.46 – 98.13 |
| NA | 96.59 – 97.87 | 97.44 – 97.65 | 96.45 – 97.51 |
| M | 98.07 – 98.57 | 98.47 – 98.78 | 98.17 – 98.57 |
| NS | 97.14 – 97.85 | 97.73 – 97.97 | 96.78 – 97.49 |
