# Supplementary material for: Rapid evolution of Mexican H7N3 highly pathogenic avian influenza viruses in poultry
Source: PLoS One. 2019 Sep 12;14(9):e0222457. doi: 10.1371/journal.pone.0222457 (PMC6742402; doi:10.1371/journal.pone.0222457)
Supplement: S3 Table — (DOCX) [file pone.0222457.s007.docx]

Supplementary Table 3. N-glycosylation site prediction in HA proteins.

| Strain | Mature HA amino acid number  (H7 numbering) | | | | | | | | | | | |
| --- | --- | --- | --- | --- | --- | --- | --- | --- | --- | --- | --- | --- |
|  | 30  (12) | 46  (28) | 141  (123) | 151  (133) | 167  (149) | 172  (154) | 182  (164) | 223  (205) | 249  (231) | 421  (403) | 493  (475) | Cluster |
| A/chicken/Jalisco_CPA1/2012 | NGTK | NATE |  |  |  |  |  |  | NDTV | NWTR | NNTY | - |
| A/Mexico/InDRE7218/2012 | NGTK | NATE |  |  |  |  |  |  | NDTV | NWTR | NNTY | - |
| A/chicken/Jalisco/12283/2012 | NGTK | NATE |  |  |  |  |  |  | NDTV | NWTR | NNTY | - |
| A/fighting_bird/Oaxaca/CPA_06257/2015 | NGTK | NATE | NGTT | NPSF | NATF |  |  |  | NDTV | NWTR | NNTY | - |
| A/chicken/Puebla/CPA_28973/2015 | NGTK | NATE | NGTT | NPSF | NATF |  |  |  | NDTV | NWTR | NNTY | - |
| A/chicken/Puebla/CPA_07421/2015 | NGTK | NATE | NGTT | NPSF | NATF |  |  |  | NDTV | NWTR | NNTY | - |
| A/backyard_poultry/Jalisco/CPA_37905/2015 | NGTK | NATE | NGTT | NPSF | NATF |  | NKSA |  | NDTV | NWTR | NNTY | A1 |
| A/chicken/Guanajuato/07437_15/2015 | NGTK | NATE | NGTT | NPSF | NATF |  | NKSA |  | NDTV | NWTR | NNTY | A1 |
| A/chicken/Guanajuato/CPA_02921_16_CENASA_95294/2016 | NGTK | NATE | NGTT | NPSF | NATF |  | NKSA |  | NDTV | NWTR | NNTY | A1 |
| A/chicken/Jalisco/716/2017 | NGTK | NATE |  | NPSF | NATF |  | NKSA | NPSP | NDTV | NWTR | NNTY | A1 |
| A/chicken/Jalisco/7LG/2017 | NGTK | NATE | NGTT | NPSF | NATF |  |  |  | NDTV | NWTR | NNTY | A1 |
| A/chicken/Jalisco/7DIEGO/2017 | NGTK | NATE | NGTT | NPSF | NATF |  | NKSA |  | NDTV | NWTR | NNTY | A1 |
| A/chicken/Jalisco/CPA_04173_16_CENASA_95294/2016 | NGTK | NATE | NGTT | NPSF | NATF |  | NKSA |  | NDTV | NWTR | NNTY | A1 |
| A/chicken/Puebla/CPA_04451_16_CENASA_95294/2016 | NGTK | NATE | NGTT | NPSF | NATF |  | NKSA |  | NDTV | NWTR | NNTY | A2 |
| A/chicken/Puebla/CPA_03191_16_CENASA_95076/2016 | NGTK | NATE | NGTT | NPSF | NATF |  | NKSA |  | NDTV | NWTR | NNTY | A2 |
| A/chicken/Puebla/CPA_04760_16_CENASA_95294/2016 | NGTK | NATE | NGTT | NPSF | NATF |  | NKSA |  | NDTV | NWTR | NNTY | A2 |
| A/chicken/Puebla/CPA_02457_16_CENASA_95294/2016 | NGTK | NATE | NGTT | NPSF | NATF |  | NKSA |  | NDTV | NWTR | NNTY | A2 |
| A/chicken/Puebla/CPA_03309_16_CENASA_95076/2016 | NGTK | NATE | NGTT | NPSF | NATF |  | NKSA |  | NDTV | NWTR | NNTY | A2 |
| A/chicken/Puebla/CPA_04148_16_CENASA_95294/2016 | NGTK | NATE | NGTT | NPSF | NATF |  | NKSA |  | NDTV | NWTR | NNTY | A2 |
| A/chicken/Jalisco/CPA_01864_16_CENASA_95294/2016 | NGTK | NATE | NGTT |  | NATF |  |  |  | NDTV | NWTR | NNTY | B |
| A/chicken/Jalisco/CPA_01859_16_CENASA_95294/2016 | NGTK | NATE | NGTT |  | NATF |  |  |  | NDTV | NWTR | NNTY | B |
| A/chicken/Jalisco/CPA_01863_16_CENASA_95294/2016 | NGTK | NATE | NGTT |  | NATF |  |  |  | NDTV | NWTR | NNTY | B |
| A/chicken/Jalisco/CPA_01858_16_CENASA_95294/2016 | NGTK | NATE | NGTT |  | NATF |  |  |  | NDTV | NWTR | NNTY | B |
| A/chicken/Jalisco/CPA_01655/2016 | NGTK | NATE | NGTT |  | NATF |  |  |  | NDTV | NWTR | NNTY | B |
| A/chicken/Jalisco/CPA_01861_16_CENASA_95294/2016 | NGTK | NATE | NGTT |  | NATF |  |  |  | NDTV | NWTR | NNTY | B |
| A/Chicken/Jalisco/PAVX17170/2017 | NGTK | NATE |  |  | NATF | NMTR |  |  | NDTV | NWTR | NNTY | B |
