# Supplementary material for: Rapid evolution of Mexican H7N3 highly pathogenic avian influenza viruses in poultry
Source: PLoS One. 2019 Sep 12;14(9):e0222457. doi: 10.1371/journal.pone.0222457 (PMC6742402; doi:10.1371/journal.pone.0222457)
Supplement: S4 Table — (DOCX) [file pone.0222457.s008.docx]

Supplementary Table 4. N-glycosylation site prediction in the NA proteins.

| Strain | Amino acid number | | | | | | | | |
| --- | --- | --- | --- | --- | --- | --- | --- | --- | --- |
|  | 14 | 57 | 66 | 72 | 146 | 308 | 341 | 457 | Cluster |
| A/chicken/Jalisco_CPA1/2012 | NTTL | NCSD | NNTI | NITT | NGTI | NETI |  |  | - |
| A/Mexico/InDRE7218/2012 | NTTL | NCSD | NNTI | NITT | NGTI | NETI |  |  | - |
| A/chicken/Jalisco/12283/2012 | NTTL | NCSD | NNTI | NITT | NGTI | NETI |  |  | - |
| A/fighting_bird/Oaxaca/CPA_06257/2015 | NTTL | NCSD | NNTI | NITT | NGTI | NETI |  |  | - |
| A/chicken/Puebla/CPA_28973/2015 | NTTL |  | NNTI | NITT | NGTI | NETI |  | NWSD | - |
| A/chicken/Puebla/CPA_07421/2015 | NTTL |  | NNTI | NITT | NGTI | NETI |  | NWSD | - |
| A/backyard_poultry/Jalisco/CPA_37905/2015 | NTTL | NCSD | NNTI | NITT | NGTI | NETI |  | NWSD | A1 |
| A/chicken/Guanajuato/07437_15/2015 | NTTL | NCSD | NNTI | NITT | NGTI | NETI | NVSG | NWSD | A1 |
| A/chicken/Guanajuato/CPA_02921_16_CENASA_95294/2016 | NTTL | NCSD | NNTI | NITT | NGTI | NETI |  | NWSD | A1 |
| A/chicken/Jalisco/716/2017 | NTTL | NCSD | NNTI | NITT | NGTI | NETI |  | NWSD | A1 |
| A/chicken/Jalisco/7LG/2017 | NTTL | NCSD | NNTI | NITT | NGTI | NETI |  | NWSD | A1 |
| A/chicken/Jalisco/7DIEGO/2017 | NTTL | NCSD | NNTI | NITT | NGTI | NETI |  | NWSD | A1 |
| A/chicken/Jalisco/CPA_04173_16_CENASA_95294/2016 | NTTL |  | NNTI | NITT | NGTI | NETI |  | NWSD | A2 |
| A/chicken/Puebla/CPA_04451_16_CENASA_95294/2016 | NTTL |  | NNTI | NITT | NGTI | NETI |  | NWSD | A2 |
| A/chicken/Puebla/CPA_03191_16_CENASA_95076/2016 | NTTL |  | NNTI | NITT | NGTI | NETI |  | NWSD | A2 |
| A/chicken/Puebla/CPA_04760_16_CENASA_95294/2016 | NTTL |  | NNTI | NITT | NGTI | NETI |  | NWSD | A2 |
| A/chicken/Puebla/CPA_02457_16_CENASA_95294/2016 | NTTL |  | NNTI | NITT | NGTI | NETI |  | NWSD | A2 |
| A/chicken/Puebla/CPA_03309_16_CENASA_95076/2016 | NTTL |  | NNTI | NITT | NGTI | NETI |  | NWSD | A2 |
| A/chicken/Puebla/CPA_04148_16_CENASA_95294/2016 |  |  | NNTI | NITT | NGTI | NETI |  | NWSD | B |
| A/chicken/Jalisco/CPA_01864_16_CENASA_95294/2016 |  |  | NNTI | NITT | NGTI | NETI |  | NWSD | B |
| A/chicken/Jalisco/CPA_01859_16_CENASA_95294/2016 |  |  | NNTI | NITT | NGTI | NETI |  | NWSD | B |
| A/chicken/Jalisco/CPA_01863_16_CENASA_95294/2016 |  |  | NNTI | NITT | NGTI | NETI |  | NWSD | B |
| A/chicken/Jalisco/CPA_01858_16_CENASA_95294/2016 |  |  | NNTI | NITT | NGTI | NETI |  | NWSD | B |
| A/chicken/Jalisco/CPA_01655/2016 |  |  | NNTI | NITT | NGTI | NETI |  | NWSD | B |
| A/chicken/Jalisco/CPA_01861_16_CENASA_95294/2016 |  |  | NNTI | NITT | NGTI | NETI |  | NWSD | B |
