# Supplementary material for: Rapid evolution of Mexican H7N3 highly pathogenic avian influenza viruses in poultry
Source: PLoS One. 2019 Sep 12;14(9):e0222457. doi: 10.1371/journal.pone.0222457 (PMC6742402; doi:10.1371/journal.pone.0222457)
Supplement: S5 Table — (DOCX) [file pone.0222457.s009.docx]

| Segment | Wild | Poultry |
| --- | --- | --- |
| PB2 | 6.71 | 5.10 |
| PB1 | 8.63 | 5.70 |
| PB1-F2 | 9.51 | 9.66 |
| PA | 8.64 | 5.93 |
| HA | 7.17 | 4.25 |
| NP | 7.90 | 8.90 |
| NA | 7.87 | 7.37 |
| M1 | 7.90 | 8.90 |
| M2 | >100 | 3.41 |
| NS1 | 5.76 | 7.17 |
| NS2 | 14.05 | 5.31 |

Supplementary Table 5. Maximum likelihood estimate of transition-to-transversion ratio
