# Supplementary material for: Rapid evolution of Mexican H7N3 highly pathogenic avian influenza viruses in poultry
Source: PLoS One. 2019 Sep 12;14(9):e0222457. doi: 10.1371/journal.pone.0222457 (PMC6742402; doi:10.1371/journal.pone.0222457)
Supplement: S6 Table — (DOCX) [file pone.0222457.s010.docx]

Supplementary Table 6. Amino acid distribution of the North American AIV for the positively selected sites in the Mexican H7N3 HPAI virus

| Segment | Amino acid changes of positively selected sites | | Amino acid distribution (%) in the North American AIV | |
| --- | --- | --- | --- | --- |
|  |  |  | Wild birds | Poultry  (chicken, turkey, ostrich, quail, and guinea fowl) |
| PB2  Poultry  (n=622)  Wild bird  (n=7538) | K116R | K  R | 99.6  0.4 | 88.1  11.8 |
|  | R389K | R  K | 98.1  1.9 | 95.7  4.3 |
| PB1  Poultry  (n=786)  Wild bird  (n=7648) | S216I | S  I  G | 96.8  0.0  1.1 | 93.1  1.7  3.8 |
| PA  Poultry  (n=636)  Wild bird  (n=7592) | D396Y | D  Y  N  G | 98.2  0.0  1.0  0.6 | 96.5  0.8  1.9  0.2 |
|  | I668V | I  V | 99.8  0.2 | 98.7  1.3 |
| HA  Poultry  (n=281)  Wild bird  (n=600 | G52K/E/R | G  K/E/R | 99.7  0.3 | 93.2  6.8 |
|  | D73K/N/S | D  K/N/S | 90.3  5.2 | 21.4  68.0 |
|  | R130T | R  T  K | 94.0  2.5  3.3 | 27.2  53.0  16.8 |
|  | G133S/N | G  S/N | 99.5  0.3 | 91.8  7.5 |
|  | K157Q/M/R | K  Q/M/R | 99.2  0.8 | 90.0  6.4 |
|  | Q201L | Q  L  R | 99.0  0.5  0.5 | 88.6  6.1  4.3 |
| NA  Poultry  (n=52)  Wild bird  (n=807) | N249S | N  S | 97.8  1.7 | 64.7  33.3 |
|  | V397M | V  M  K | 99.9  0.0  0.1 | 73.1  17.3  0.0 |
|  | K412R | K  R  S  E | 83.9  2.4  0.0  10.9 | 86.5  7.7  3.8  0.0 |
| M2  Poultry  (n=525)  Wild bird  (n=5493) | S23N | S  N | 99.4  0.6 | 97.3  2.7 |
| NS1  Poultry  (n=251)  Wild bird  (n=4040) | A112T | A  T  I | 98.7  1.1  0.0 | 86.1  2.8  11.2 |
|  | D171G/N | D  G/N | 98.9  0.7 | 86.5  8.8 |
|  | V180T | V  T  G | 99.0  0.0  0.8 | 90.0  8.0  1.2 |
